# Diversification events and the effects of mass extinctions on Crocodyliformes evolutionary history

**DOI:** 10.1098/rsos.140385

**Published:** 2015-05-27

**Authors:** Mario Bronzati, Felipe C. Montefeltro, Max C. Langer

**Affiliations:** 1 Bayerische Staatssammlung für Paläontologie und Geologie, Richard-Wagner-Strasse 10, Munich 80333, Germany; 2 Ludwig-Maximilians Universität, Munich, Germany; 3 Faculdade de Filosofia Ciências e Letras de Ribeirão Preto, University of Sao Paulo, Avenida Bandeirantes 3900, 14040–901 Ribeirão Preto, São Paulo, Brazil; 4 Departamento de Biologia e Zootecnia, Faculdade de Engenharia de Ilha Solteira, UNESP, Rua Monção 226, 15385-000 Ilha Solteira, São Paulo, Brazil

**Keywords:** Crocodyliformes, diversification, phylogeny, topology-based methods, mass extinction

## Abstract

The rich fossil record of Crocodyliformes shows a much greater diversity in the past than today in terms of morphological disparity and occupation of niches. We conducted topology-based analyses seeking diversification shifts along the evolutionary history of the group. Our results support previous studies, indicating an initial radiation of the group following the Triassic/Jurassic mass extinction, here assumed to be related to the diversification of terrestrial protosuchians, marine thalattosuchians and semi-aquatic lineages within Neosuchia. During the Cretaceous, notosuchians embodied a second diversification event in terrestrial habitats and eusuchian lineages started diversifying before the end of the Mesozoic. Our results also support previous arguments for a minor impact of the Cretaceous/Palaeogene mass extinction on the evolutionary history of the group. This argument is not only based on the information from the fossil record, which shows basal groups surviving the mass extinction and the decline of other Mesozoic lineages before the event, but also by the diversification event encompassing only the alligatoroids in the earliest period after the extinction. Our results also indicate that, instead of a continuous process through time, Crocodyliformes diversification was patchy, with events restricted to specific subgroups in particular environments and time intervals.

## 2. Introduction

Archosauria is one of the most successful radiations of vertebrates, with 250 Myr of evolutionary history since its origin during the Triassic [1]. The group is composed of two lineages, the avemetatarsalians, including birds and their non-avian dinosaur forerunners, and the pseudosuchians, including the living crocodiles [1]. The Triassic–Jurassic mass extinction (approx. 200 Ma) did not significantly reduce Avemetatarsalia diversity, but it was devastating for pseudosuchians, with Crocodylomorpha as the only group crossing the Triassic boundary [2]. Crocodylomorpha includes the ‘sphenosuchians’, a series of small, terrestrial predators restricted to Late Triassic to Late Jurassic rocks [3], and the Crocodyliformes, encompassing most of the past diversity of the group, as well as the living representatives [4]. The group not only survived the first event of mass extinction that followed its origin (the Triassic–Jurassic event) but also surpassed the Cretaceous–Palaeogene (K/Pg) boundary (approx. 66 Ma). Its astonishing fossil record, with representatives found worldwide, shows an incredible variety of forms and niche occupation.

Crocodyliformes appear in the fossil record in the Late Triassic of Argentina [5], approximately 220 Ma, but *Hemiprotosuchus leali* remains the only Triassic crocodyliform known so far. The basal ‘protosuchians’ embody an array of small- to medium-sized fully terrestrial lineages that most likely represent a paraphyletic group relative to Mesoeucrocodylia [6], which includes most of Crocodyliformes subgroups (e.g. Notosuchia, Thalattosuchia, Dyrosauridae and the crown-group Crocodylia). Most ‘protosuchian’ lineages are restricted to the Jurassic, whereas others extend its occurrence into the Cretaceous [6]. ‘Protosuchians’ temporally coexisted with the thalattosuchians, a remarkable group known from Jurassic to Cretaceous marine deposits [7]. Members of Thalattosuchia show extreme adaptations to the marine environment [8], and its phylogenetic position is one of the most controversial among crocodyliforms [7,9,10]. The notosuchians, another group of terrestrial crocodiles, shows a great diversity of size and feeding habits [11,12]. The basal members of this group are found in Cretaceous rocks of Gondwana [13], with one possible exception in Asia [12]. However, if sebecids are placed within Notosuchia, the group extends its occurrence to Cenozoic deposits [14]. Based on dentition and jaw morphology, some notosuchians are inferred to be omnivorous or herbivorous [15]. Other notosuchians, such as baurusuchids, were carnivorous and considered top predators in the terrestrial faunas of South America during the Late Cretaceous (approx. 100–66 Ma), reaching up to 4 m in length [16].

A higher diversity is not restricted to the basal lineage of Crocodyliformes, some fossil members of the crown-group Crocodylia also deviate from the typical body plan of living crocodiles. Some examples are the small bodied, brevirostrine with bulbous crushing teeth *Allognatosuchus*, from Laurasian deposits from the Eocene of North America [17], and the huge flat-headed *Mourasuchus*, from the Miocene of South America [18].

It is a widespread idea that Crocodyliformes was much more diverse in the past, as suggested by the apparent great morphological disparity of its fossil representatives [19]. Even if the morphological discrepancy among living crocodilians is somehow underestimated [20], it is undisputed that fossil members of the group occupied a much broader niche [7,12,21]. In vertebrate palaeontology, different approaches have been taken to address diversity changes along geological time (e.g. [2,22–26]). Here, we employed a topology-based method to identify diversification shifts along the entire crocodyliform evolutionary history. This new category of data provides additional support for a great diversification event following the Triassic–Jurassic mass extinction [27]; and new data suggesting a small-scale impact of the K/Pg mass extinction in the Early Cenozoic diversification of the group. Also, this study points out the diversification events which led Crocodyliformes, represented by less than 30 species in modern environments, to achieve such astonishing diversity in the past.

## 3. Material and methods

### 3.1 Phylogenetic framework

The diversification analyses conducted here are based on an updated version of the supertree of Crocodyliformes [28] which was built by translating information of previous phylogenetic analyses of the group into a MRP-Matrix ([29,30], and see [28]). A heuristic search of the resulting data matrix was conducted in TNT [31] (10 000 replicates, hold 20, and TBR—branch swapping). The strict consensus of the MPT's retrieved low resolution and we employed the *IterPCR* script for TNT [32] to identify unstable taxa. These were excluded from the original data matrix and a new analysis was performed with the same parameters. Also, as in the original study [28], the supertree was built with Crocodylia summarized in a terminal branch. To avoid the loss of information regarding the diversity of the crown-group, that terminal branch was replaced by the topology resulting from a reanalysis of Brochu [33]. We first re-ran the data matrix of the original paper using TNT (10 000 replicates, hold 20, TBR—branch swapping). As in the original study, the resulting topology of our analyses also exhibited a series of unresolved nodes. The *IterPCR* script was also employed and a new analysis excluding unstable taxa performed. Taxa for which phylogenetic information in the context of the crown-group phylogeny were not taken into account in the supertree matrix had their position established based on the Crocodylia phylogeny derived from this latter analysis. The final combined topology contains 245 taxa that encompass most of the known past and present diversity of Crocodyliformes. The topology was employed as the main framework for the following diversification analyses (see the electronic supplementary material for additional information about the topology).

### 3.2 Diversification analyses

Diversification analyses were conducted in the software SymmeTree [34]. The inclusion of non-coexistent taxa in SymmeTree (i.e. simultaneous inclusion of fossil and extant taxa) violates one of the premises of the Yule evolution model [35] used as the null hypothesis. In order to tackle this problem, pioneer studies dealing with fossil taxa in SymmeTree [36,37] applied a time slice procedure. This procedure breaks the entire geological history of the group into defined time intervals in which all taxa coexisted. Each time slice contains only the taxa occurring in that period plus the ghost lineages inferred from earlier periods (see [36]).

Tarver & Donoghue [38] argue that the methodology of Ruta *et al.* [36] does not violate the Yule model, but it does not overcome the inability to distinguish between true diversification shifts, associated to speciation, and shifts recovered because of the extinction of older lineages. Tarver & Donoghue [38] proposed the nested-growth method as an alternative to the time-slicing procedure. In this approach, each time slice encompasses not only the taxa found on the interval and the inferred ghost lineages of younger taxa, but also the taxa from previous time slices. In this way, the tree grows from the oldest to the most recent time interval and, as taxa are added and never excluded, it simulates the effect of speciation, but not extinction. One advantage of the nested-grown methodology in comparison to previous approaches is that the former gives the possibility to discriminate genuine events of diversification from artefacts. For instance, in cases where the fossil record supports an older origin than the detected shift, it might be related to extinction of ancient lineages and not solely on the appearance of new ones. When facing this particular scenario, we considered as genuine only the diversification shifts in which older taxa does not exceed 10% of the group species richness on the interval when the shift is first detected. By using this approach, we imply that a group diversification is not always synchronic with its origin in the geological record and that a group can experience radiations even relatively later after its origin.

Analyses were performed using 10 000 random resolutions per node and 1 000 000 for the entire tree. Diversifications were identified based on the *Δ*_2_ shift statistic. Analyses were performed for 10 different trees representing distinct time intervals, following the nested-growing methodology of Tarver & Donoghue [38]. The time intervals used were: 1. Carnian—Aalenian; 2. Bajocian—Oxfordian; 3. Kimmeridgean—Barremian; 4. Aptian—Albian; 5. Cenomanian—Santonian; 6. Campanian—Maastrichtian; 7. Palaeocene; 8. Eocene; 9. Oligocene—Miocene; and 10. Pliocene—Recent. Ages of taxa were obtained from published sources. Taxa with records in different time slices or with uncertain ages were scored based on the oldest record/age.

## 4. Results

**Figure 1.**
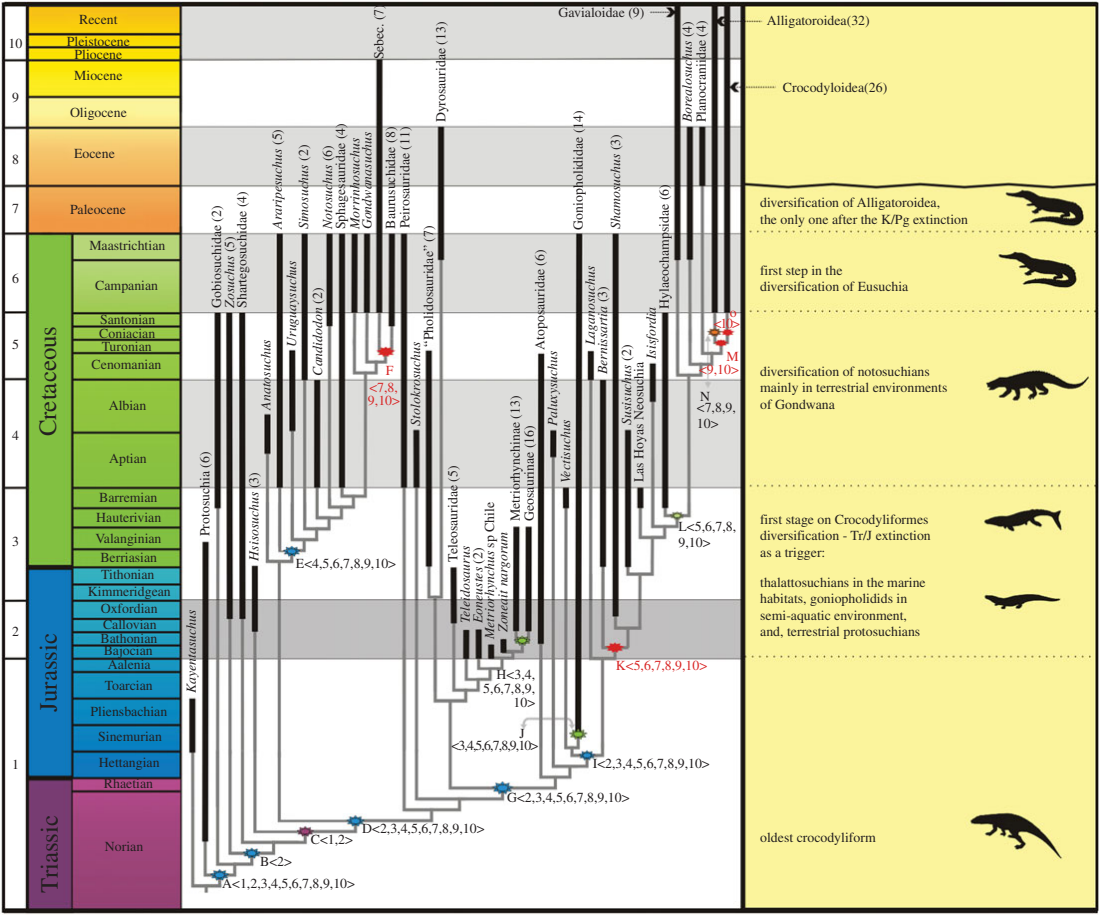
Summary of the Crocodyliformes phylogeny depicting diversification events. Each terminal taxon has its fossil record, represented by black lines, depicted against geological time. Clades are collapsed and numbers in parentheses represent the total number of terminal taxa within each branch (some clades are represented by the name of higher level groups and others by the name of a genus within the clade). The node related to clades for which diversification shifts were obtained in the analyses are marked—the colour assigned to the symbol matches the colour of the period related to the shift and red was assigned to those interpreted as artefacts. Numbers within < > correspond to the intervals on which these shifts were identified. The ages of cladogenetic events, within the grey lines, do not correspond to time intervals in which they are depicted on the figure. Names of clades related to shifts identified in the analysis—A: Crocodyliformes; B: unnamed clade (all taxa more closely related to *Zosuchus* than to *Gobiosuchus*); C: unnamed clade (*Hsisosuchus*+Mesoeucrocodylia); D: Mesoeucrocodylia; E: Notosuchia; F: Sebecosuchia; G: Neosuchia; H: Metriorhynchidae; I: unnamed clade (all taxa more closely related to *Goniopholis* than to *Theriosuchus*); J: Goniopholididae; K: unnamed clade (all taxa more closely related to *Bernissartia* than to *Laganosuchus*); L: Eusuchia; M: Brevirostrines; N: Alligatoroidea; O: Crocodyloidea.

The shifts detailed below are assigned to the same clade through the analysis but the composition of each clade expands successively at each time bin because of the nested-grown methodology (see the electronic supplementary material for more details). A shift for Crocodyliformes was recovered in time bin 1 (figure 1—node A). Diversification shifts for Mesoeucrocodylia (figure 1—node D), Neosuchia (figure 1—node G) and the unnamed clade that encompass all taxa more closely related to *Goniopholis* than to *Paluxysuchus* (including Eusuchia) were recovered in time bin 2 (figure 1—node I). In time bin 3, diversification shifts for Goniopholididae (figure 1—node J) and Metriorhynchidae (figure 1—node H) were detected. A shift for Notosuchia (figure 1—node E) was recovered in time bin 4. In time bin 5, the shifts for Eusuchia (figure 1—node L) and to the unnamed clade formed by all taxa more closely related to *Bernissartia* than to *Laganosuchus* (figure 1—node K) were obtained. No new diversification shifts were identified in time interval 6. A shift within Notosuchia, for Sebecosuchia (figure 1—node F), and a shift for Alligatoroidea (figure 1—node N) were recovered in time bin 7. Also within the crown-group Crocodylia, other shifts identified are the ones for Brevirostrine (figure 1—node M) in time bin 9 and for Crocodyloidea (figure 1—node O) in time bin 10.

The shifts detailed above were recovered in all subsequent time intervals. Other shifts do not show the same pattern and are recovered punctually in some time range. A diversification shift for the clade composed of *Hsisosuchus*+Mesoeucrocodylia (figure 1—node C) was recognized in time bins 1 and 2, but not in the subsequent time intervals. Another shift, related to an unnamed clade containing all taxa more closely related to *Zosuchus* than to *Gobiosuchus* (figure 1—node B), was identified only in time bin 2 (see the electronic supplementary material).

## 5. Discussion

We did not take the diversification shifts obtained in SymmeTree as direct evidence of radiation of lineages. Instead, we used a twofold approach, which also acknowledges the fossil record of Crocodyliformes in order to distinguish between genuine events and putative artefacts (*sensu* [38]). However, differences on the quality of the rock sampling through geological time also affects the output of the biological signal from the fossil record [39,40]. This phenomenon hampers the direct reading of the fossil record as a fully reliable source of the past diversity of tetrapod groups [26,41–44]. In the context of the nested-grown methodology, it is conceivable that a well-sampled time period is misinterpreted as a diversification shift if preceded by a not well-sampled one. However, at this moment, there is no available tool to deal with rock sample bias in this tree-based approach and future studies with a probabilistic approach might overcome such problems [38].

Another potential bias of our analyses relies on the topological framework, as our topology cannot at the same time address dramatic alternative hypotheses found in the literature. Examples of extreme incongruence in previous studies are related to taxa such as thalattosuchians, which were placed either as basal mesoeucrocodylians or neosuchians [7,9,10]; *Araripesuchus*, previously assigned as a basal Notosuchia or closer to Peirosauridae [7,45–47]; and the sebecids and baurusuchids forming Sebecosuchia or sebecids closer to peirosaurids and forming Sebecia [14,21,47–50]. The supertree approach, which is better interpreted as a ‘synthetic consensus’ (*sensu* [36]) of the phylogenetic knowledge of a group, represents one of the possibilities to deal with alternative topologies. In our study, we only address the thalattosuchian problem in our discussion because its number of taxa (more than 40) has the greater potential to affect the outcome of the nested-grown methodology. As potentially for all groups of organisms, Crocodyliformes phylogeny is and eventually will always be in state of flux, and future studies should take into account the new data (e.g. [51,52]).

### 5.1 The initial diversification of Crocodyliformes

The analyses show a diversification shift at the branch leading to Crocodyliformes (figure 1—node A) in the Late Triassic—Early Jurassic interval (time bin 1) but the geometric shape of phylogenetic trees tends to favour the recovery of shifts in the basal dichotomy of the tree [37]. In order to have a more robust evidence for this shift, we conducted an exploratory analysis in an alternative scenario with a sequence of outgroup taxa added accordingly to the phylogeny presented in [1]. The diversification shift in Crocodyliformes is still recovered, even when the clade no longer derives from the basal dichotomy, suggesting that it corresponds to a true diversification. Time bin 1 includes the Late Triassic; however, as the fossil record known from this period for Crocodyliformes is restricted to *H. leali*, an Early Jurassic age for the event is more likely (an additional analysis restricting time bin 1 to the Late Triassic was also conducted and no shift was identified). Results of disparity analyses [27] also suggest that this was a key period in the diversification of the crocodylomorphs. This assumption strongly relies on the record of thalattosuchians and the goniopholidid *Calsoyasuchus valliceps* in the Early Jurassic [53]. Indeed, their current position in the phylogeny implies that most crocodyliform lineages were already present at that time. Even if thalattosuchians are not found as one of the branches of the Mesoeucrocodylia basal dichotomy [47], the consensual position of Goniopholididae within Neosuchia [54] holds the Early Jurassic age for the ghost lineages of most subgroups [7]. Combining the results of our analyses with the information from the Early Jurassic record for Crocodyliformes, we dismember this radiation in two events related to different crocodyliform groups. The ‘protosuchians’, that at this time already had its diversity close to the totality of the group, are associated with the shift at the base of Crocodyliformes. In Mesoeucrocodylia, however, the diversification is preferably assigned to more derived subgroups (i.e. Neosuchia), which are recorded at this interval, whereas typically Cretaceous groups such as Notosuchia and Peirosauridae are not. Our results seem to support that Crocodyliformes diversification is in fact related to the Triassic–Jurassic extinction, which was previously identified as a trigger for Crocodylomorpha radiation [27]. The discovery of new fossils in Jurassic rocks can strength this scenario, bringing more evidence for the diversification of the group at this period.

### 5.2 Further Mesozoic diversification

Diversification shifts recovered in time bin 2 (Bajocian–Oxfordian) are better interpreted as continuations and subdivisions of the shifts recognized in the previous time bin for derived Crocodyliformes, and confirms that these clades have been already radiating since deeper times in crocodyliform history. Most of the diversity known from this interval is related to taxa highly adapted to the aquatic environment, the goniopholidids and the marine thalattosuchians. Indeed, the high diversity of Bajocian–Oxfordian thalattosuchians, especially metriorhynchids [55], is a major influence for the detection of the shifts in this time bin. As the position of Thalattosuchia is still controversial [7,9,10], an exploratory analysis using an alternative position for the group was performed depicting the group as one of the branches connected at the basal Mesoeucrocodylia dichotomy. The diversification shift at the base of Neosuchia (figure 1—node G) for time bin 2 (recovered in the original analyses) was not detected. Instead, a shift appears at the branch leading to the Mesoeucrocodylia clade (figure 1—node D). Moreover, the shifts related to Metriorhynchidae (figure 1—node H), Goniopholididae (figure 1—node J) and ‘pro-Eusuchia’ lineages are also recovered for time bin 2 in both analyses. In this context, instead of relating this shift to the base of Neosuchia or to the Mesoeucrocodylia clade, we connected the shift to the radiation of the less inclusive metriorhynchids, and neosuchians, including derived taxa closer to Eusuchia and Goniopholididae. Metriorhynchidae and Goniopholididae include taxa with Early Jurassic occurrences (time bin 1) but most of the lineages appear later in the Middle Jurassic [54,55], indicating that shifts related to these lineages are not artefacts caused by extinction of earlier lineages. The metriorhynchid diversity shifts detected in this time bin partially agrees with a previous claim for a ‘diversity burst’ for the clade during the Kimmeridgian–Tithonian driven by the variation on the sea level and the sea surface temperature [56]. Both methodologies are probably detecting the same biological signal but the different bases of them lead to divergences on the interpretation of the age of the evolutionary event.

The Cretaceous high diversity of Crocodyliformes is mostly related to the terrestrial notosuchian. Diversification shifts related to that group (figure 1—node E) were identified from time bin 4 (Aptian–Albian) onwards. This interval encompasses the Aptian, the oldest period in which notosuchians are undoubtedly recognized in the fossil record—*Malawisuchus mwakasyungutiensis* [57] and *Pakasuchus kapilimai* [58] (but see [13]). Given the congruence between the fossil record and the results of the diversification analyses, we consider this as a genuine shift. Nevertheless, it is worth mentioning that the detection of such shift for Notosuchia, as well as our current understanding of the early evolution of the group as taking place mainly in the Aptian of Gondwana, might be dramatically challenged with new discoveries from the poor and patchily sampled Early Cretaceous fossil record [26]. On the other hand, this is not the case for another shift within Notosuchia, identified at time bin 7 (Palaeocene) and related to the Sebecosuchia clade (figure 1—node F). The group has a broad fossil record in the Cenozoic, achieving the status of major predators in South America at that time [50]. However, baurusuchids, which represent a large portion of sebecosuchian diversity, and the basal notosuchians became extinct at the end of the Cretaceous [58]. The extinction of these taxa seems to strongly bias the shift recovered for Sebecosuchia, which is here considered as an artefact. Nevertheless, the affinity of Sebecosuchia to Notosuchia remains uncertain [50] and the status of this shift may change in alternative phylogenetic scenarios (e.g. [47]).

Regarding the only two shifts first identified in Cenomanian—Santonian (time bin 5), one is related to the clade composed by taxa more closely related to *Bernissartia* than to *Laganosuchus* (figure 1—node K). The comparison to the fossil record shows that most of the taxa encompassed in this diversity shift actually correspond to the same components of the earlier radiation of neosuchians, with a minor contribution of ghost lineages of younger clades. This scenario indicates that the shift for this group is an artefact produced by the extinction of older lineages. The other shift recovered in time bin 5 is detected within Eusuchia (Hylaeochampsidae plus Crocodylia, excluding *Isisfordia*—figure 1—node L) and is here treated as a genuine event. *Hylaeochampsa* pulls back the origin of the Hylaeochampsidae clade to the Early Cretaceous (Barremian) but other putative hylaeochampsids (*Pietraroiasuchus*, *Acynodon*), *Borealosuchus* and ghost lineages of Crocodylia subgroups are seen in the interval associated to time bin 5 (Cenomanian—Santonian). The shift implies a Cenomanian—Santonian radiation for Eusuchia including Crocodylia ghost-lineages, but crown-group lineages only became abundant in the Cenozoic after their origin in the latest Cretaceous [59].

### 5.3 Diversification after the Cretaceous–Palaeogene event

With respect to the Crocodylia crown-group, a shift related to Alligatoroidea starting in the Palaeocene (figure 1—node N), time bin 7, is here considered a genuine diversification event. Alligatoroidea first appeared in the Campanian, based on the record of *Stangerochampsa* and *Brachychampsa* [20], but the Cretaceous fossil record is minimal when compared with the Cenozoic record of the group. On the other hand, the diversification shift related to Crocodyloidea+Alligatoroidea (Brevirostrines—figure 1—node M) does not appear in the first stages of the Cenozoic, but later in the Miocene. The Miocene is a period of recognized diversification of crocodyloidea lineages [22,60], and indeed, the shift detected in the analysis encompass the vast fossil record of the group. However, as the shift was not exclusively related to the Crocodyloidea lineage, but to the broader clade Brevirostrines, we treated the shift as an artefact influenced by the Early Cenozoic extinction of lineages, some of them related to the Palaeocene shift identified for Alligatoroidea (see the electronic supplementary material).

The Alligatoroidea diversification shift is the only one found in the time interval immediately after the end-Cretaceous mass extinction. The existence of this shift has implications for the understanding of the impact of that extinction event in the evolutionary history of Crocodyliformes. Lineages that were abundant during the Mesozoic, such as thalattosuchians, and goniopholidids were either extinct or in decline during the Late Cretaceous [7,54,58], which is reflected in the absence of new shifts in this time interval. The fossil record also shows that not only lineages of the crown-group crossed the Mesozoic/Cenozoic barrier, and even if not associated to significant diversification shifts, the marine dyrosaurids and terrestrial sebecids had some diversity in the Early Cenozoic [50,61]. In this way, the fossil record indicates that the decline of Mesozoic lineages was not mostly driven by the K/Pg extinction [62], some perished before and some lasted still long after the event. Also, this extinction has not triggered a great diversification event in the Early Cenozoic evolution of Crocodyliformes, being restricted to a single group, contrary to the scenario after the Triassic–Jurassic extinction on which shifts encompass most lineages present at that moment. Finally, the survival and diversity of non-Eusuchia lineages is evidence against a causal relationship between the extinction of these forms in the K/Pg boundary and the diversification of the crown-group in the early stages of Cenozoic, as hypothesized by Markwick [22].

## 6. Conclusion

This study combined the statistical support of topology-based analyses to identify diversification shifts with direct information from the fossil record to bring new data to the study of the crocodyliform evolutionary history. A previous disparity study [27] proposed that the initial radiation of Crocodylomorpha took place along the Early Jurassic, and our results also place the early diversification of the slightly less inclusive Crocodyliformes in that period of time. These evidences from two distinct methodological studies indicate that the Early Jurassic was an important moment in the establishment of the crocodyliform lineage, however such radiation is hidden by the scarcity of fossils from this epoch. Aditionally, Mesozoic crocodyliforms succeeded in environments and/or areas not fully explored by non-avian dinosaurs. After the initial radiation in the Late Triassic [37], dinosaurs were still prevalent and continued diversifying in the terrestrial habitats of the Early Jurassic [63]. During this latter period, Crocodyliformes diversifications occurred mainly in those habitats not explored by its archosaur relatives, as exemplified by the diversification of marine thalattosuchians and neosuchian lineages in marine and semi-aquatic environments during the Jurassic/Early Cretaceous. The posterior Cretaceous diversification of Notosuchia encompassing omnivorous/herbivorous forms occurred mainly in parts of Gondwana, where carnivorous dinosaurs and mammals seem to have played a secondary palaeoecological role [16,58]. After a peak during the Early Cretaceous, the fossil record shows a general decline in Mesoeucrocodylia diversity towards to the K/Pg event [7,58], whereas Eusuchia lineages diversified. Not only lineages of the Crocodylia crown-group survived to the extinction [22,59]. Dyrosaurids [61] and sebecosuchians [50] were still diverse in marine and terrestrial environments respectively, but the Cenozoic diversification event is only related to Crocodylia subgroup. Nevertheless, the appearance of distinct crocodylian lineages during the Cenozoic [60] was not enough to counterbalance the general decline in diversity of the group in modern ecosystems, a scenario probably driven by changes in the global temperature [22].

Overall, the analyses performed herein indicate that the incredible fossil diversity of crocodyliforms was not achieved via a continuous process through time, encompassing the entire group, but was generated by diversification events in a small number of ecologically diverse clades occurring in restricted time intervals. Groups related to the initial diversification after the Triassic–Jurassic extinction, such as ‘protosuchians’, thalattosuchians and goniopholidids, did not continue to diversify during their entire range along the Mesozoic. Also, later events such as the diversification of Notosuchia in the Cretaceous and of Alligatoroidea in the Palaeocene are not extensions of previous events, but represent isolated cases of subgroup diversification restricted to particular time intervals. Regarding the two mass extinctions that the group endured, they impacted markedly differently on the two major lineages of Archosauria. Dinosaurs started diversifying earlier, in terrestrial habitats during the Carnian–Norian ([37,63,64], but see [65]), before the mass extinction event. On the other hand, the Triassic–Jurassic extinction was a trigger for the diversification of Crocodyliformes, which started occupying habitats and areas not fully explored by its closest relatives. The impact of the K/Pg mass extinction in non-avian dinosaur diversity is well known [66], but that event did not greatly affect the evolutionary history of Crocodyliformes.

## Supplementary Material

1. ESM main document - Describe the procedures of the phylogenetic and diversification analyses

## Supplementary Material

2. Crocodyliformes phylogeny - Supertree Crocodyliformes data matrix

## Supplementary Material

3. Crocodyliforme phylogeney - Supertree Crocodyliformes data matrix after excluding the unstable taxa 4. Crocodylia phylogeny - Data matrix for the phylogenetic analysis of the crown group 5. Crocodylia phylogeny modified - Data matrix for the phylogenetic analysis of the crown group after excluding unstable taxa 6 - Diversification analysis results

## Data Accessibility

The datasets supporting this article have been uploaded as part of the electronic supplementary material.
