# Supplementary material for: Diversification events and the effects of mass extinctions on Crocodyliformes evolutionary history
Source: R Soc Open Sci. 2015 May 27;2(5):140385. doi: 10.1098/rsos.140385 (PMC4453258; doi:10.1098/rsos.140385)
Supplement: 1. ESM main document - Describe the procedures of the phylogenetic and diversification analyses [file rsos140385supp1.docx]

Electronic Supplementary Material

**Diversification events and the effects of mass extinctions on Crocodyliformes evolutionary history**

Mario Bronzati^a,b,c*^, Felipe C. Montefeltro^d^, Max C. Langer^c^

^a^ Bayerische Staatssammlung für Paläontologie und Geologie, Richard-Wagner-Straße 10, 80333 Munich, Germany.

^b^ Ludwig-Maximilians Universität, Munich, Germany

^c^ Faculdade de Filosofia Ciências e Letras de Ribeirão Preto, USP, Av. Bandeirantes 3900, 14040-901 Ribeirão Preto, São Paulo, Brazil.

^d^ Departamento de Biologia e Zootecnia, Faculdade de Engenharia de Ilha Solteira, UNESP, Rua Monção 226 , Ilha Solteira, São Paulo, Brazil.

*Author for correspondence (m.bronzati@lrz.uni-muenchen.de)

| **This file includes:** |  |
| --- | --- |
| 1. List of publications containing the source trees added to the updated version of the MRP matrix of Bronzati et al. 2012 …………..…………........................... 02 2. Crocodyliformes Supertree and Crocodylia phylogeny ………………………. 04 3. Diversification analysis…..…………………………………………………… 14 4. Additional references ………………………………………………………….. 31 |  |

**1 - List of publications containing the source trees added to the updated version of the MRP matrix of Bronzati et al. 2012**

Adams, T. L. (2013). A new neosuchian crocodyliform from the Lower Cretaceous (late Aptian) Twin Mountains Formation of North-Central Texas. *Journal of Vertebrate Paleontology* 33: 85–10

Andrade, M. B., Edmonds, R., Benton, M. J.; Schouten, R. (2011), A new Berriasian species of *Goniopholis* (Mesoeucrocodylia, Neosuchia) from England, and a review of the genus. *Zoological Journal of the Linnean Society*, 163:S 66–S108

Brochu, C. A. (2012) A new species of *Borealosuchus* (Crocodyliformes, Eusuchia) from the Late Cretaceous-Early Paleogene of New Jersey. *Journal of Vertebrate Paleontology*. 32(1): 105-116.

Buscalioni, A. D., Piras, P., Vullo, R., Signore, M., Barbera, C. (2011), Early Eusuchia crocodylomorpha from the vertebrate-rich Plattenkalk of Pietraroia (Lower Albian, southern Apennines, Italy). *Zoological Journal of the Linnean Society*, 163: S199–S227.

Cau, A. & Fanti, F. (2011) The oldest known metriorhynchid from the Middle Jurassic of North-eastern Italy: *Neptunidraco ammoniticus* gen. et sp. nov. *Gondwana Research*. 19(2): 550-565.

Clark, J. M. (2011) A new shartegosuchid crocodyliform from the Upper Jurassic Morrison Formation of western Colorado. *Zoological Journal of the Linnean Society*, 163: S152–S172.

Figueiredo, R. G.: Moreira, J. K. R.; Saraiva, A. F. A.; Kellner, A. W. A. (2011) Description of a new specimen of *Susisuchus anatoceps* (Crocodylomorpha: Mesoeucrocodylia) from the Crato Formation (Santana Group) with comments on Neosuchia. *Zoological Journal of the Linnean Society*, 163: S273–S288

Fortier, D., Perea, D.; Schultz, C. (2011), Redescription and phylogenetic relationships of *Meridiosaurus vallisparadisi*, a pholidosaurid from the Late Jurassic of Uruguay. *Zoological Journal of the Linnean Society*, 163: S257–S272

Hastings, A. K., Bloch, J., Jaramillo, C. A. (2011). A new longirostrine dyrosaurid (Crocodylomorpha, Mesoeucrocodylia) from the Paleocene of North-eastern Colombia: biogeographic and behavioural implications for new-world dyrosauridae. *Palaeontology*. 54(4): 1095-1116.

Herrera, Y.; Gasparini, Z.; Fernández, M. S. (2013) A new Patagonian species of *Cricosaurus* (Crocodyliformes, Thalattosuchia): first evidence of *Cricosaurus* in Middle–Upper Tithonian lithographic limestone from Gondwana. *Palaeontology* 56 (3): 663–678

Holliday, C. M. & Gardner, N. M. (2012). A New Eusuchian Crocodyliform with Novel Cranial Integument and Its Significance for the Origin and Evolution of Crocodylia. *PLoSONE* 7 (1): e30471

Iori, F. V.; Marinho, T. D. S.; Carvalho, I. D. S.; Campos, A. C. D. A. (2013). Taxonomic reappraisal of the sphagesaurid crocodyliform *Sphagesaurus montealtensis* from the Late Cretaceous Adamantina Formation of São Paulo State, Brazil. *Zootaxa* 3686 (2): 183

Martin, J. E. & Buffetaut, E. (2012) The Maxillary Depression of Pholidosauridae: An Anatomical Study. *Journal of Vertebrate Paleontology* 32:6, 1442-1446

Martin, J. E.; Rabi, M.; Csiki, Z. (2010) Survival of *Theriosuchus* (Mesoeucrocodylia: Atoposauridae) in a Late Cretaceous archipelago: a new species from the Maastrichtian of Romania. *Naturwissenschaften* 97: 845-854.

Montefeltro, F.C., Larsson, H.C.E., & Langer, M.C. (2011) A New Baurusuchid (Crocodyliformes, Mesoeucrocodylia) from the Late Cretaceous of Brazil and the Phylogeny of Baurusuchidae. *PLoSONE* 6(7): e21916

Montefeltro, F. C.; Larsson, H. C. E.; França, M. A. G.; Langer, M. C. (2013). A new neosuchian with Asian affinities from the Jurassic of northeastern Brazil. *Naturwissenschaften*

Pariilla-Bel, J.; Young, M. T.; Moreno-Azanza, M.; Canudo, J. I. (2013) The first metriorhynchid Crocodylomorph from the Middle Jurassic of Spain with implications for evolution of the subclade Rhacheosaurini. *PlosOne*

Pol, D. & Powell, J. E. (2011) A new sebecid mesoeucrocodylian form the Rio Loro Formation (Paleocene) of North-western Argentina. *Zoological Journal of the Linnean Society*. 163: S7-S36.

Pritchard, A. C.; Turner, A. H.; Allen, E. R. Norell, M. A. (2013) Osteology of a North American Goniopholidid (*Eutretauranosuchus* *delfsi*) and Palate Evolution in Neosuchia. *American Museum Novitates* 3783 :1-56.

Puértolas E., Canudo, J.I., Cruzado-Caballero, P. (2011): A new crocodilian rom the Late Maastrichtian of Spain: implications or theinitial radiation of crocodyloids. *PlosONE*. (6, 6), e20011.

Nascimento, P. M. & Zaher, H. (2011) The skull of Upper Cretaceous baurusuchid crocodile *Baurusuchus albertoi* Nascimento & Zaher 2010, and its phylogenetic affinities. *Zoological Journal of the Linnean Society*. 163: S116-S131.

Riff, D. & Kellner, A. W. A. (2011) Baurusuchid crocodyliforms as theropod mimics: clues from the skull and appendicular morphology of *Stratiotosuchus maxhechti* (Upper Cretaceous of Brazil). *Zoological Journal of the Linnean Society*. 163: S37:S56.

Soto, M.; Pol, D.; Perea, D. (2011) A new specimen of *Uruguaysuchus aznarezi* (Crocodyliformes, Notosuchia) from the middle Cretaceous of Uruguay and its phylogenetic relationships. *Zoological Journal of the Linnean Society*. 163: S173-S198.

Turner, A. H. & Sertich, J. W. (2010) Phylogenetic history of *Simosuchus clarki* (Crocodyliformes: Notosuchia) from the Late Cretaceous of Madagascar". *Journal of Vertebrate Paleontology* 30 (6, Supplement): 177–236

Young M. T., Bell M. A., Andrade M. B., Brusatte S. L. (2011) Body size estimation and evolution in metriorhynchid crocodylomorphs: implications for species diversification and niche partitioning. *Zoological Journal of the Linnean Society*. 163:1199-1216

Young M. T., Bell M. A., Brusatte S. L. (2011) Craniofacial form and function in Metriorhynchidae (Crocodylomorpha: Thalattosuchia): modelling phenotypic evolution with maximum likelihood methods. *Biology* *Letters* 7: 913–916

Young, M. T.; Andrade, M. B.; Brusatte, S. L.; Sakamoto, M.; Liston, J. (2013) The oldest known metriorhynchid super-predator: a new genus and species from the Middle Jurassic of England, with implications for serration and mandibular evolution in predacious clades. *Journal of Systematic Palaeontology*. Published online

**2 – Crocodyliformes supertree and Crocodylia phylogeny**

We updated the data matrix of Bronzati *et al.* (2012) including phylogenetic analyses (item 1 – Electronic Supplementary Material) for Crocodyliformes published until the end of 2013. A first numerical analysis of the resulting data matrix (available on request) was conducted on the software TNT (Goloboff *et al*. 2009). A heuristic search (10.000 replicates, hold 20, and TBR – branch swapping) was performed and 3,539 most parsimonious trees (MPT’s) with 4,603 steps were found. As the strict consensus of the MPT’s had a low resolution (Supplement Fig. 1), we employed the *IterPCR* script (Pol & Escapa, 2009) to identify the unstable taxa in a procedure outlined in Pol & Escapa (2009) and Bronzati *et al.* (2012).

The unstable taxa identified by the IterPCR script (*Acherontisuchus guajiraensis, Araripeuschus rattoides, Barcinosuchus arveloi, Caryonosuchus pricei, Cricosaurus litographicus, Eopneumatosuchus, Eremosuchus elkoholicus, Goniopholis crassidens, Goniopholis stovalli, Iberosuchus macrodon, Leidyosuchus canadensis, Maledictosuchus riclaensis, Montsecosuchus depereti,* *Oceanosuchus* *boecensis*, *Pachycheilosuchus trinquei, Pakasuchus kapilimai, Pehuenchesuchus enderi, Peipehsuchus teleorhinus, Rhabdognathus* sp., *Steneosaurus boutilieri, Steneosaurus durobrivensis*) were excluded from the original data matrix and a new analysis was performed under the same parameters of the first one. 159 MPT’s (4,562 steps) were recovered, the majority rule consensus (Fig. 2) of which was used as the basic framework of the diversity analyses.


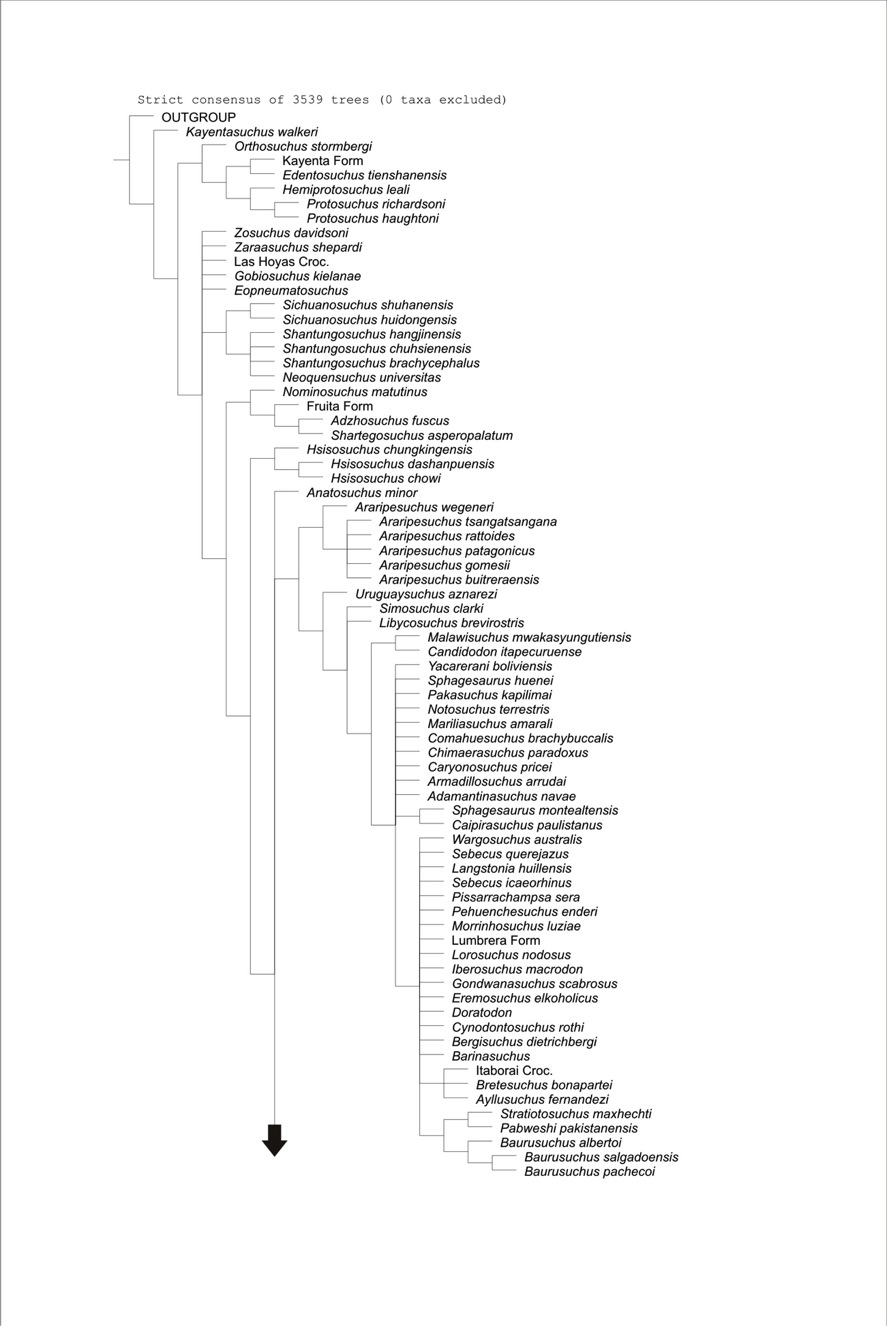

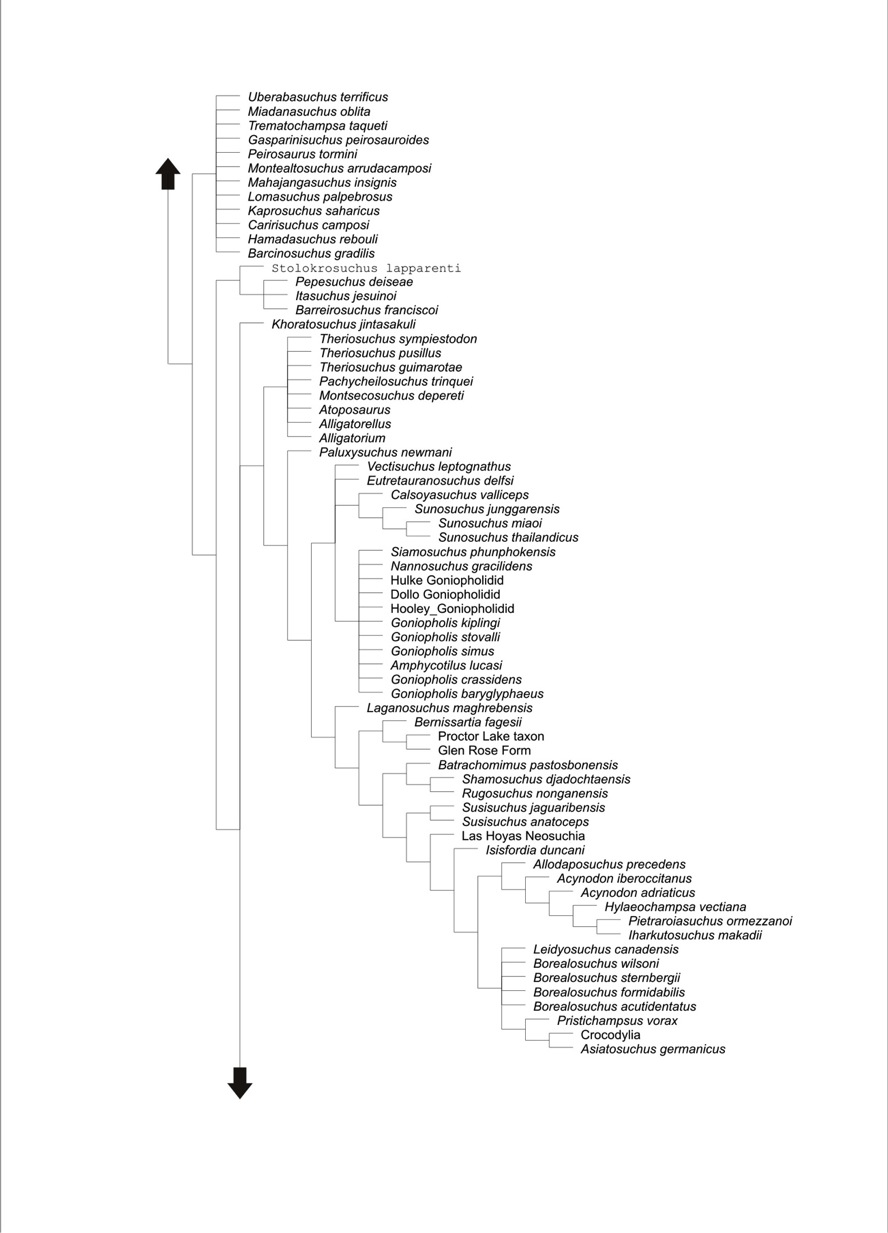

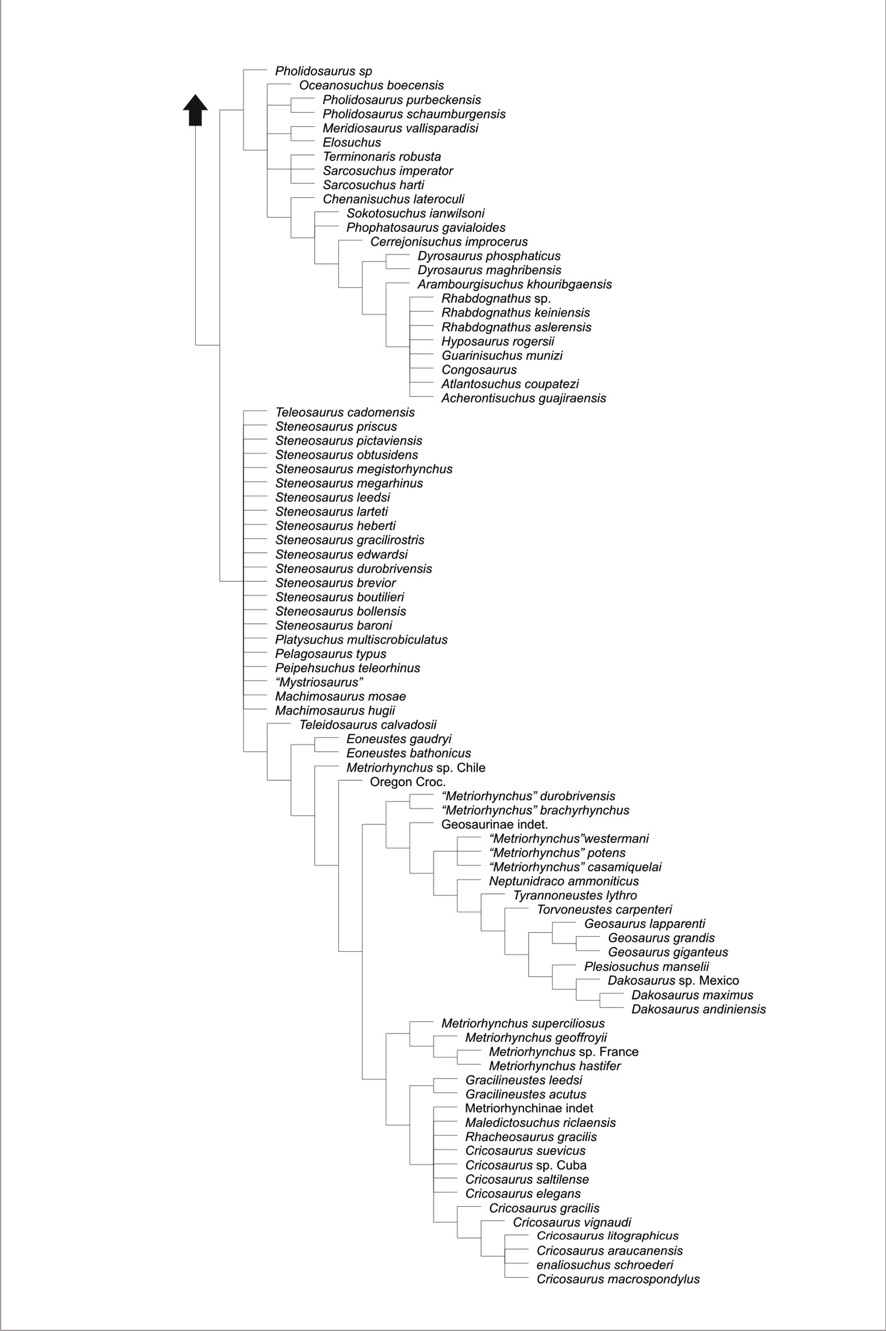


Figure 1: Strict rule consensus of the 3,593 MPT’s obtained from the first analysis of the supertree data matrix.


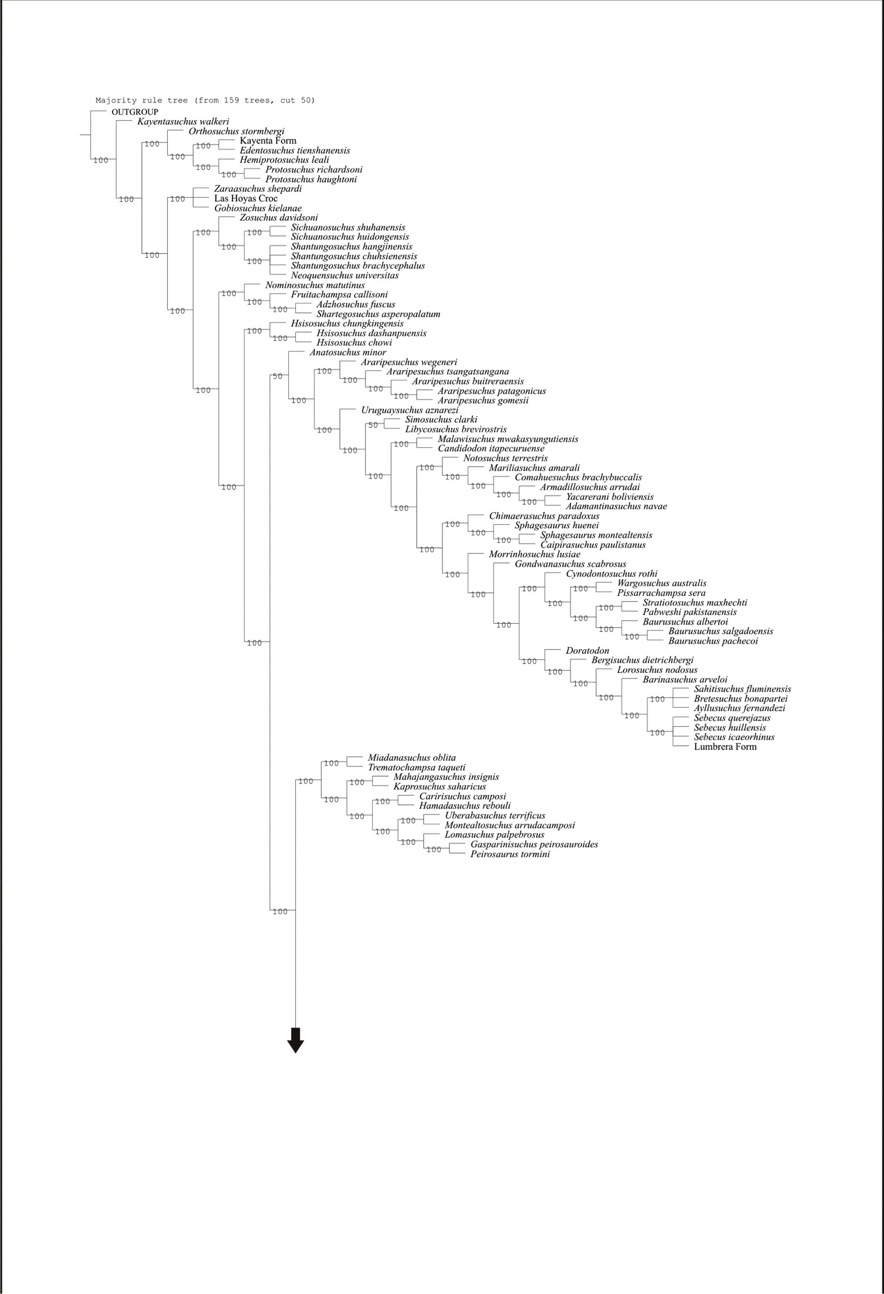


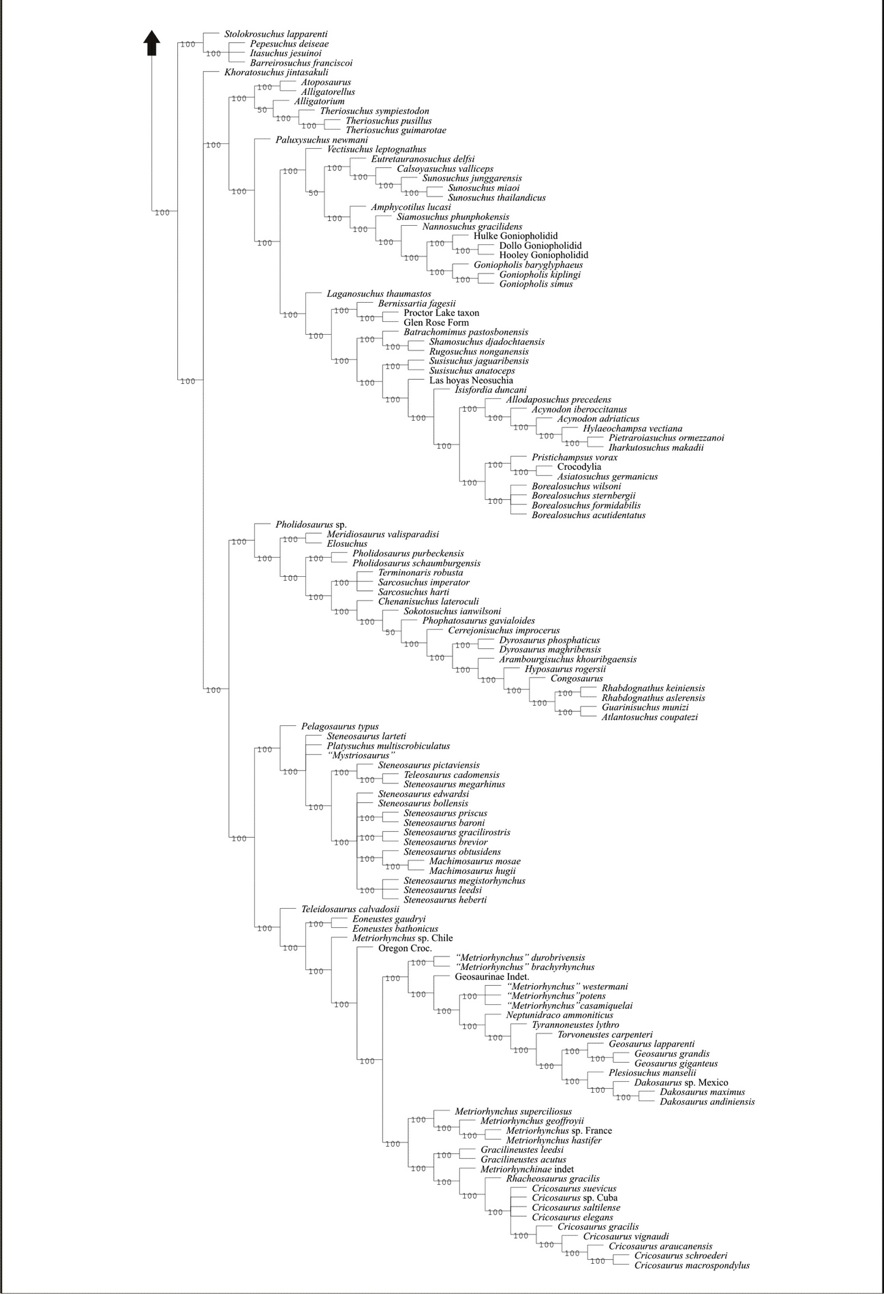


Figure 2: Majority rule consensus of the 159 MPT’s obtained in the second analysis, after excluding the most unstable taxa from the supertree data matrix.

As stated in the main text, the Crocodyliformes supertree employed here was built using Crocodylia as a terminal taxon. Accordingly, to avoid the loss of information from such a diverse clade, we replaced that terminal taxon by the phylogeny presented by Brochu (2012), which is based on one of the most recent and complete data matrix for the group. We first re-ran the data matrix of the original paper using TNT under the following parameters (10.000 replicates, hold 20, TBR – branch swapping), recovering 281 MPT’S (671 steps long). This resulted in a topology slightly distinct from that presented by Brochu et al. (2012). As in the original study, the topology has a very low resolution (Fig. 3). Accordingly, the *IterPCR* script was also used to identify the unstable taxa; which were *Asiatosuchus germanicus, Baryphracta deponiae, Hassiacosuchus haupti, Necrosuchus ionensis*, and *Tomistoma cairense.* Six MPT’s (661 steps long) were obtained from the analysis of the new matrix excluding those taxa. Their majority rule consensus (Fig. 4) was used to replace the terminal taxon Crocodylia in the supertree.


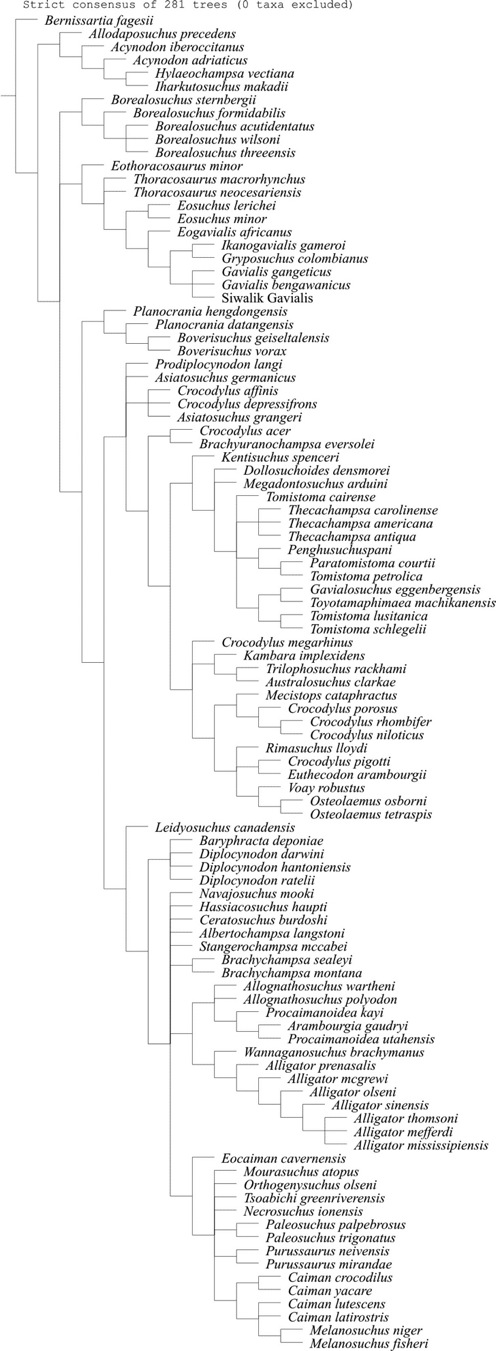


Figure 3: Strict consensus of the 281 MPT’s obtained in the first analysis using the dataset of Brochu et al. (2012).


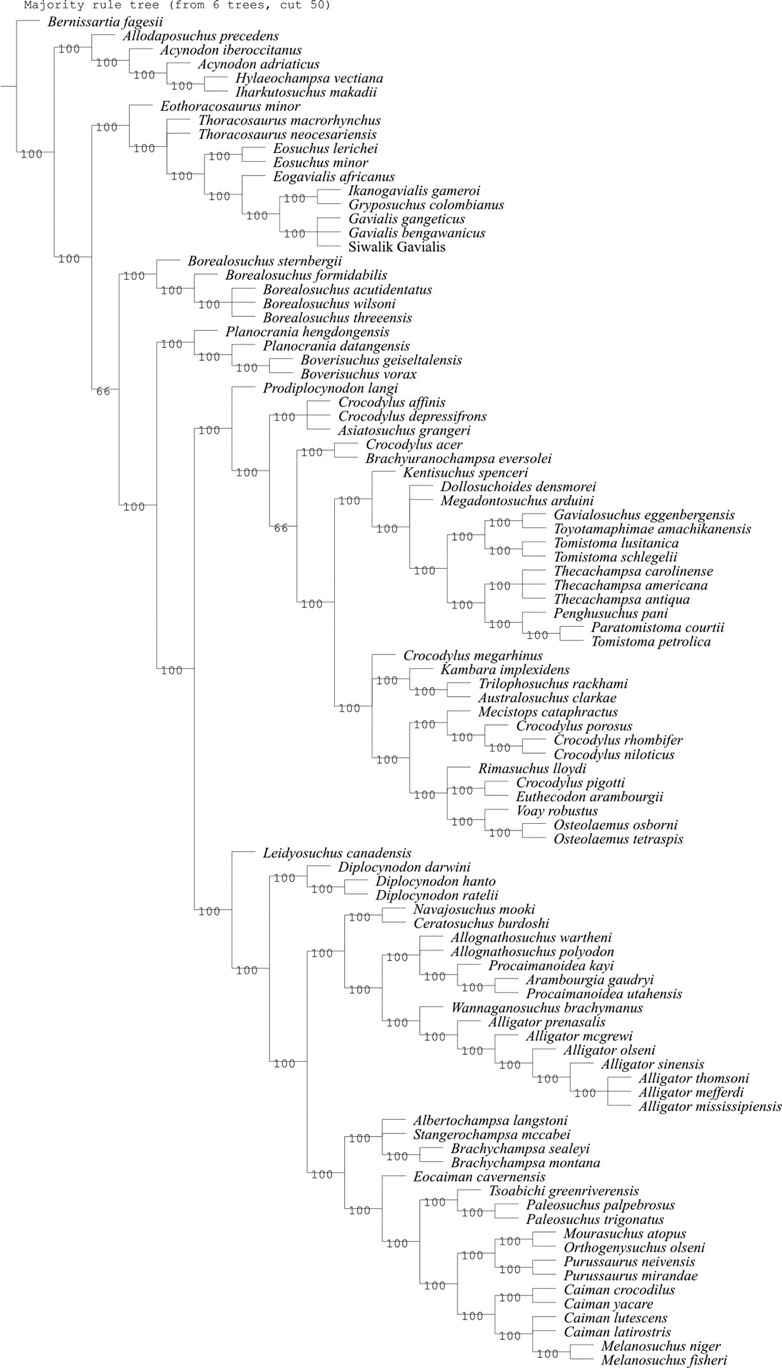


Figure 4: Majority-rule consensus of the six MPT’s obtained in the analysis of the dataset from Brochu et al. (2012) after excluding the most unstable taxa.

**Posterior Taxa prunning**

*Barreirosuchus franciscoi, Itasuchus jesuinoi, Pepesuchus deiseae* and *Carisisuchus camposi* were pruned because their placement in the supertree are not reliable. The sister group relationship of *C. camposi* and *Hamadasuchus rebouli* has no support from any of the source trees. That relation probably results from the inclusion of the study by Andrade et al. (2011), one of the few that includes *C. camposi* and the only one including this taxon simultaneously with *H. rebouli.* As these taxa are part of a highly inclusive clade, they share many nodes that likely group them together in the supertree.

In a similar case, *Barreirosuchus, Itasuchus* and *Pepesuchus* form a monophyletic sister group to *Stolokrosuchus lapparenti* in the supertree. This same relationship is proposed by Montefeltro et al. (2013), where these taxa are placed within a broader Peirosauridae clade. However, *Stolokrosuchus* has a very controversial phylogenetic position. Some analyses (e.g.: Turner & Buckley 2008, Turner & Sertich 2010) depict the taxon as more closely related to the Neosuchia than to Peirosauridae, as also seen in our supertree. However, *Pepesuchus, Itasuchus* and *Barreirosuchus* were never considered more closely related to Neosuchia than Peirosauridae. In this case, we think that the position of these taxa is strongly biased by the position of *Stolokrosuchus* and not reliable as a source of information for diversification analyses*.*

*Pristichampsus vorax, Asiatosuchus germanicus*, and *Borealosuchus* were also pruned from the supertree and positioned following the study of Brochu et al. (2012). As in Bronzati et al. (2012), this is because not all the phylogenetic information available for these taxa were employed in building of the data matrix of the supertree.

Another (latter) procedure was the pruning of some taxa in order to obtain a fully resolved topology. This was conducted because some softwares cannot deal with not fully dichotomous trees; in this way, the resulting topology used in this study can be used as a framework for different kinds of analyses. Taxa excluded in order to obtain a fully resolved topology were *Khoratosuchus jintasakuli,* Lumbrera Form, *Sahitisuchus fluminensis,* teleosaurids, *Cricosaurus suevicus, Cricosaurus* sp. Cuba, *Cricosaurus saltilense* and *Cricosaurus elegans.* Also to obtain a resolved topology, the following taxa were excluded from the Crocodylia phylogeny: “Siwalik *Gavialis”, Crocodylus depressifrons, Asiatosuchus granger, Borealosuchus threeensis Dollosuchoides densmorei, Thecachampsa antiqua, Rimasuchus lloydi, Allognatosuchus wartheni, Alligator mefferdi, Albertochampsa langstoni* and *Caiman lutescens.*

The resulting topology, after combining the Crocodyliformes Supertree with the Crocodylia phylogeny, and after the pruning procedures, is presented in the next section (i.e.: tree used in the diversification analysis for time bin 10).

**3 – Diversification analysis**

Initially, fifteen distinct time intervals were employed, ten are those used by Lloyd et al. (2008) for the Mesozoic, and five were defined for the Cenozoic. However, as the number of taxa from some intervals is very low, trees from these intervals were just slightly different from those preceding them, what leaded to a reduction in the number of time intervals. The intervals here used are: 1. Carnian – Aalenian; 2. Bajocian – Oxfordian; 3. Kimmeridgean – Barremian; 4. Aptian – Albian; 5. Cenomanian – Santonian; 6. Campanian – Maastrichtian; 7. Paleocene; 8. Eocene; 9. Oligocene – Miocene; 10. Pliocene – Recent. Age of taxa were obtained from the literature.

The trees were created according to the methodology proposed in Tarver & Donoghue (2011). Following, the resulting topologies for each one of the 10 time intervals used for the diversification analyses are depicted. The arrows are marked on the branch where diversification shift was detected in the analyses of SymmeTREE. Black arrows indicate significant shifts of diversification and white arrows indicate substantial shifts (see .xls file containing the information and the statistical background of the analyses for each one of the intervals).


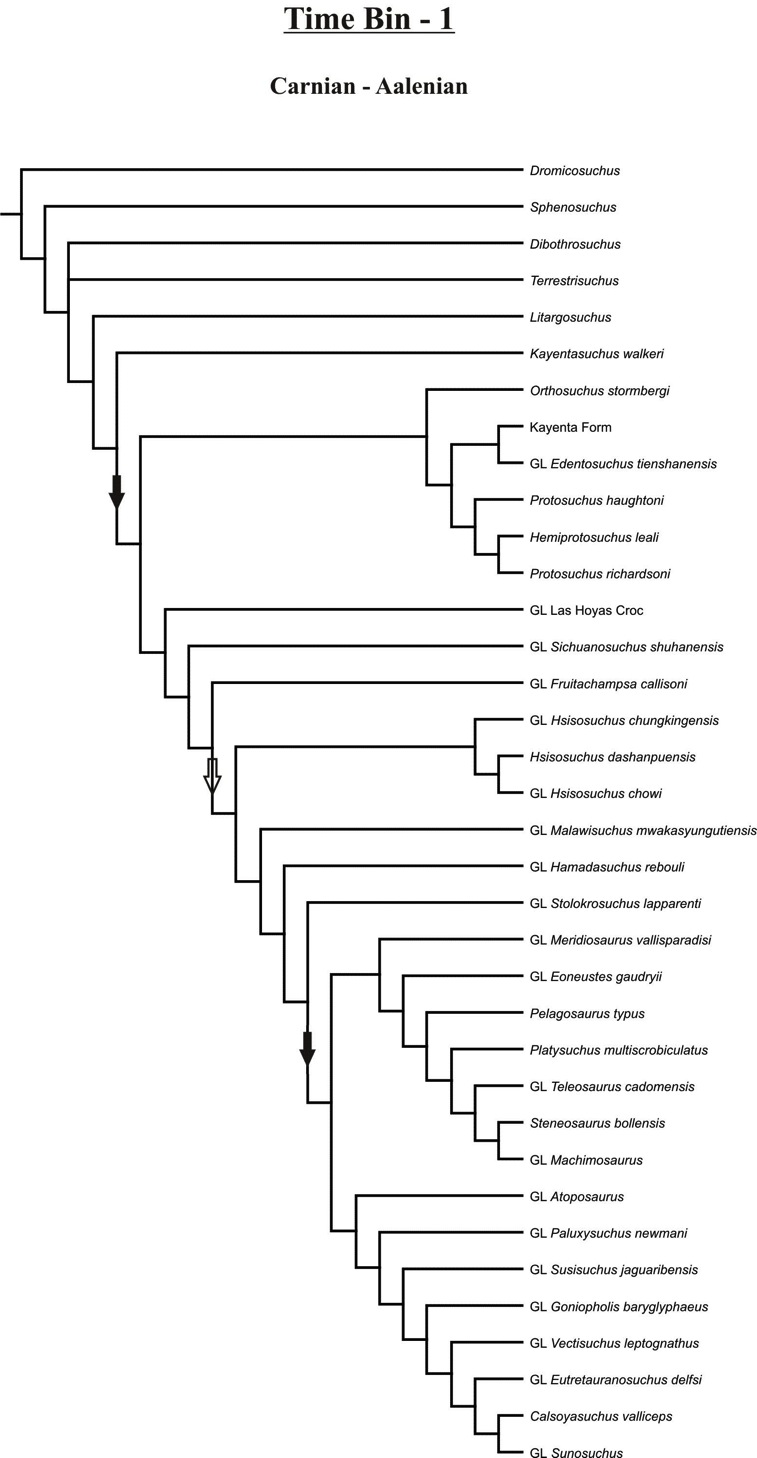

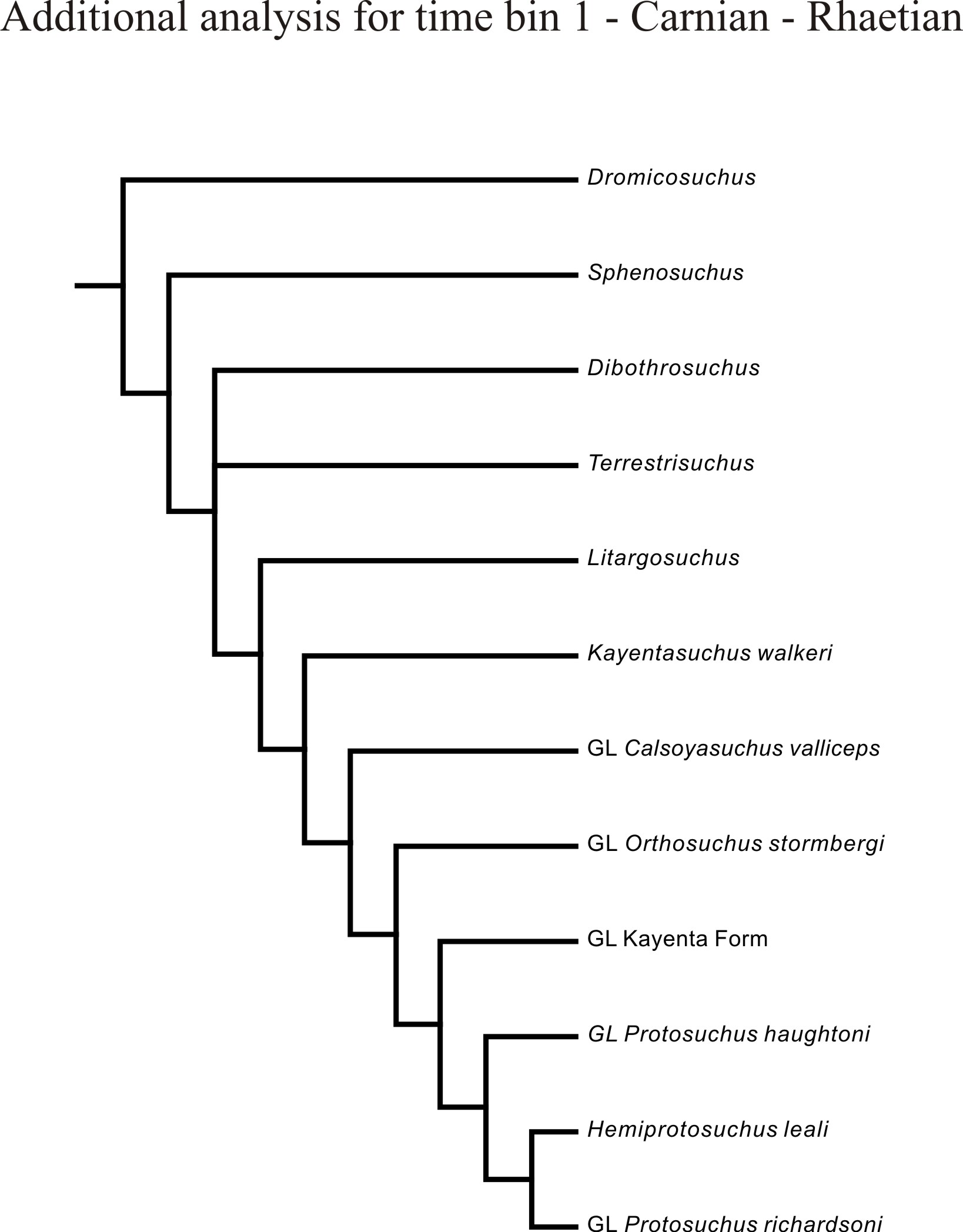

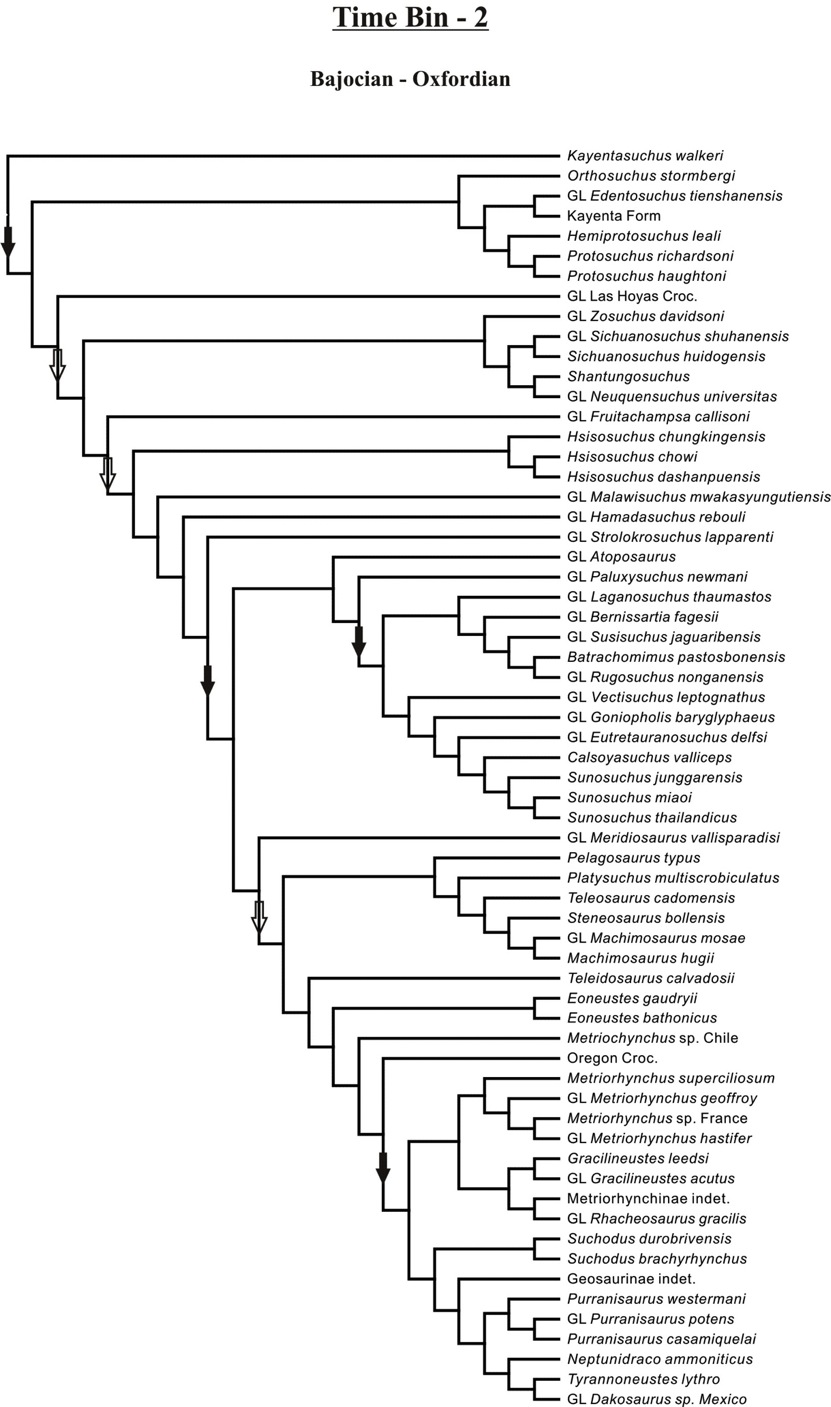


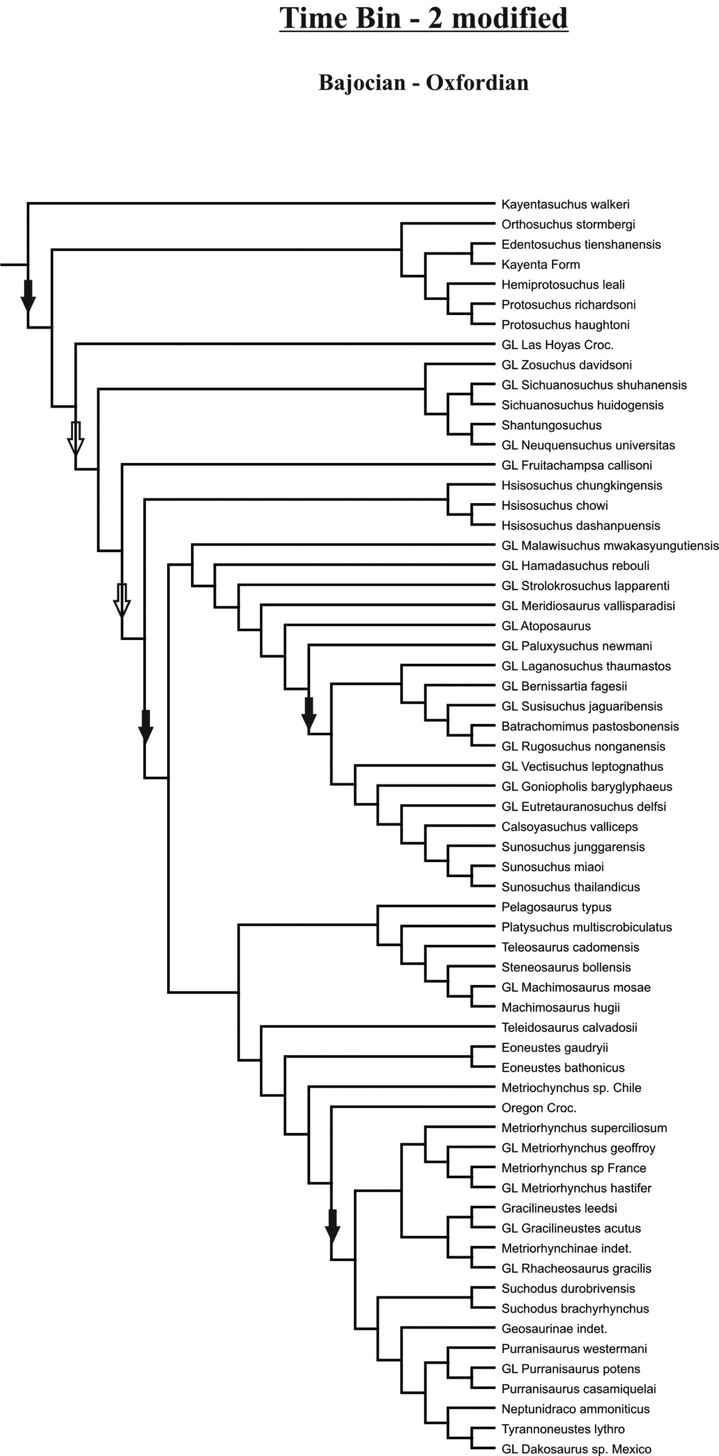

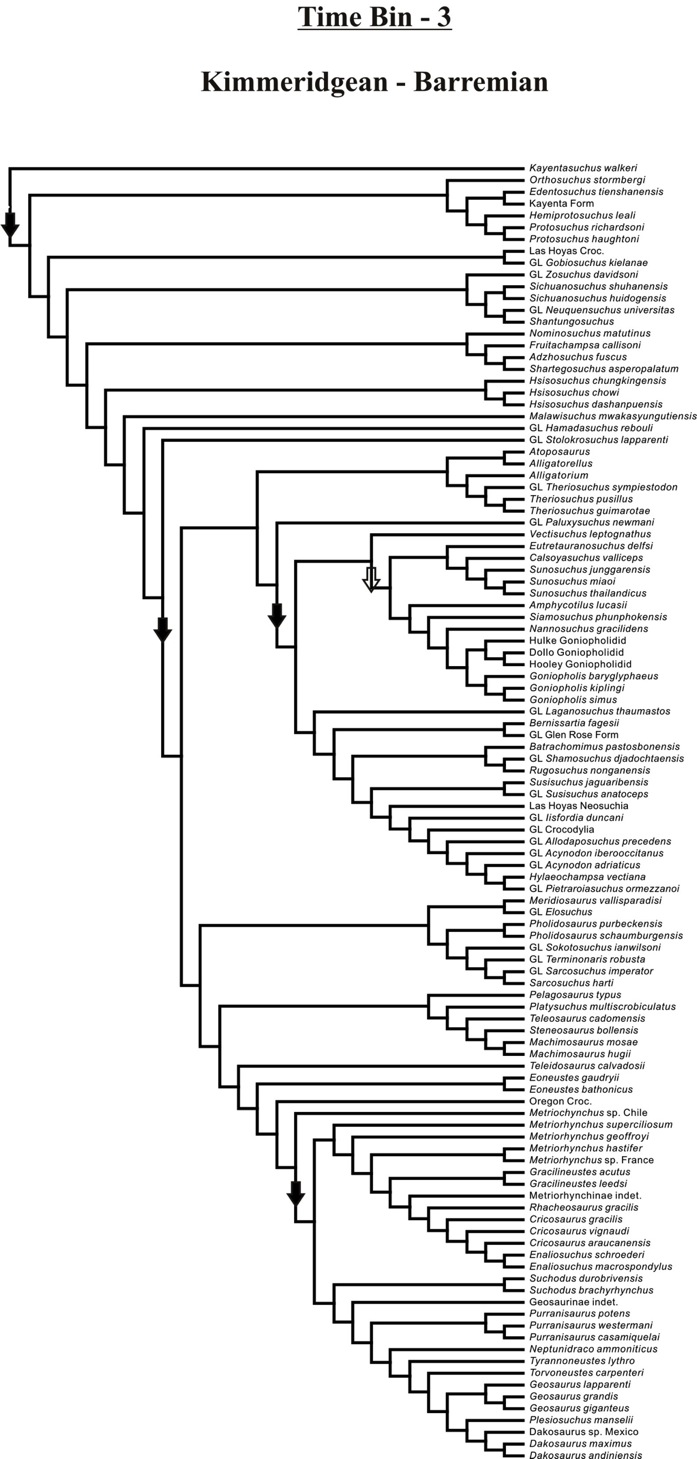

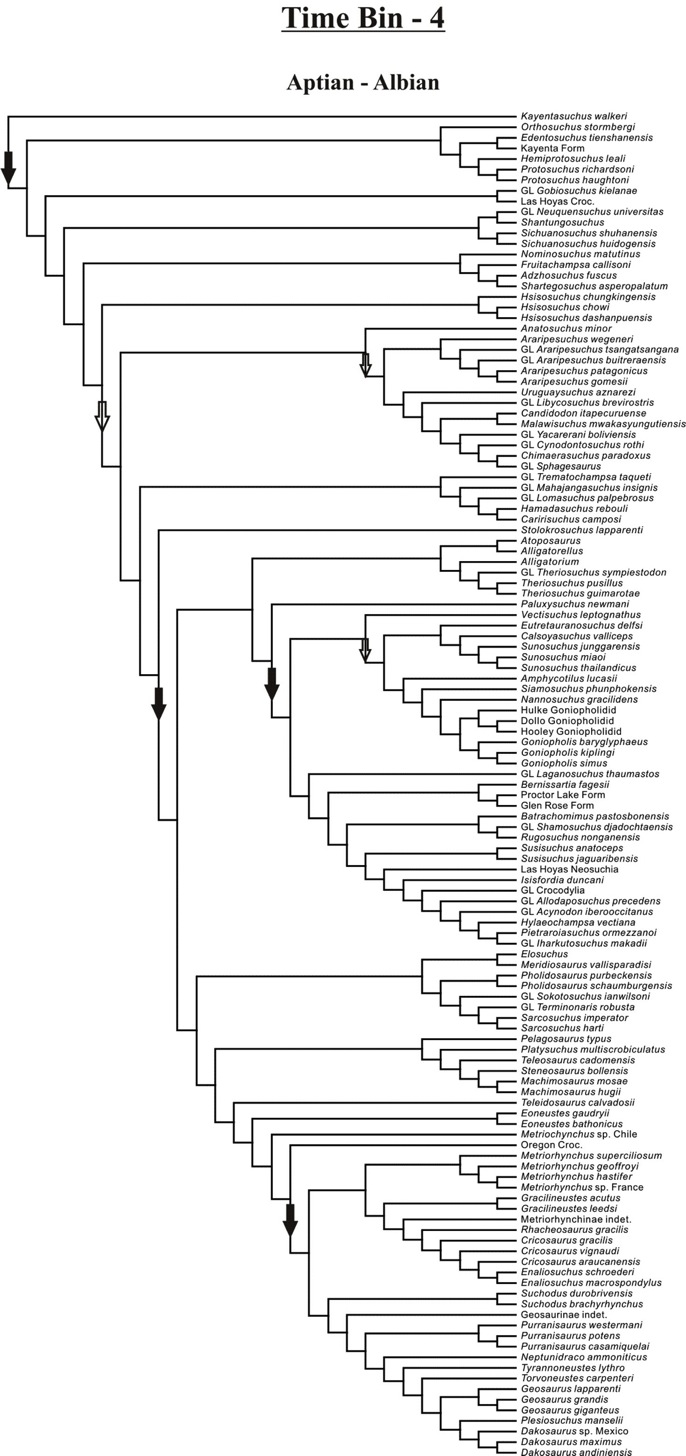

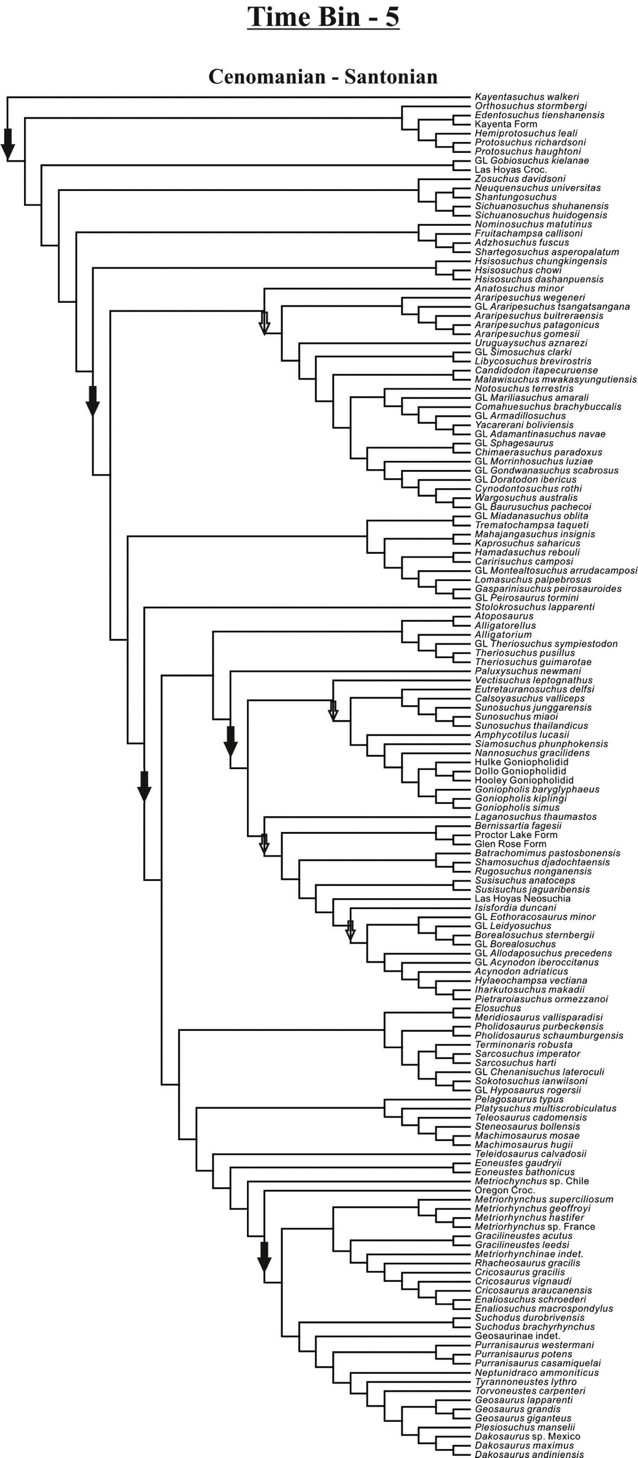

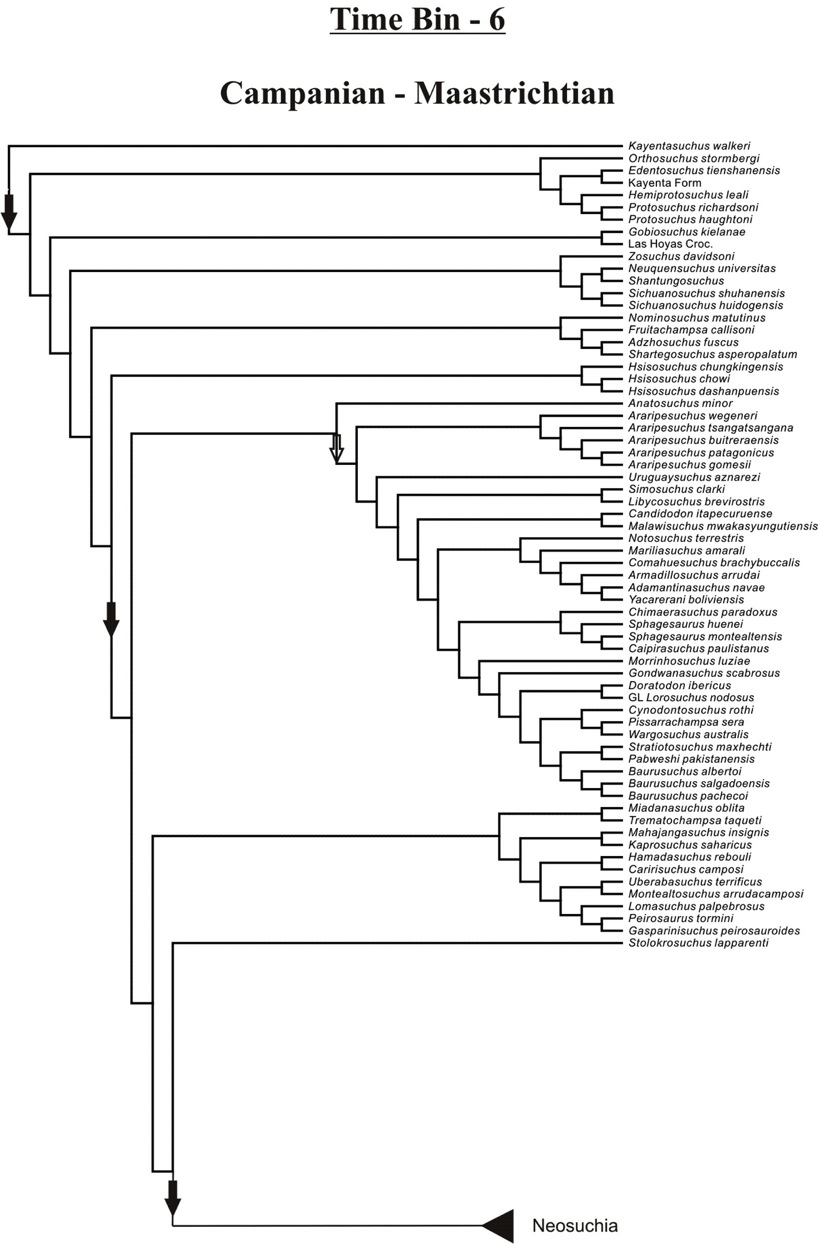

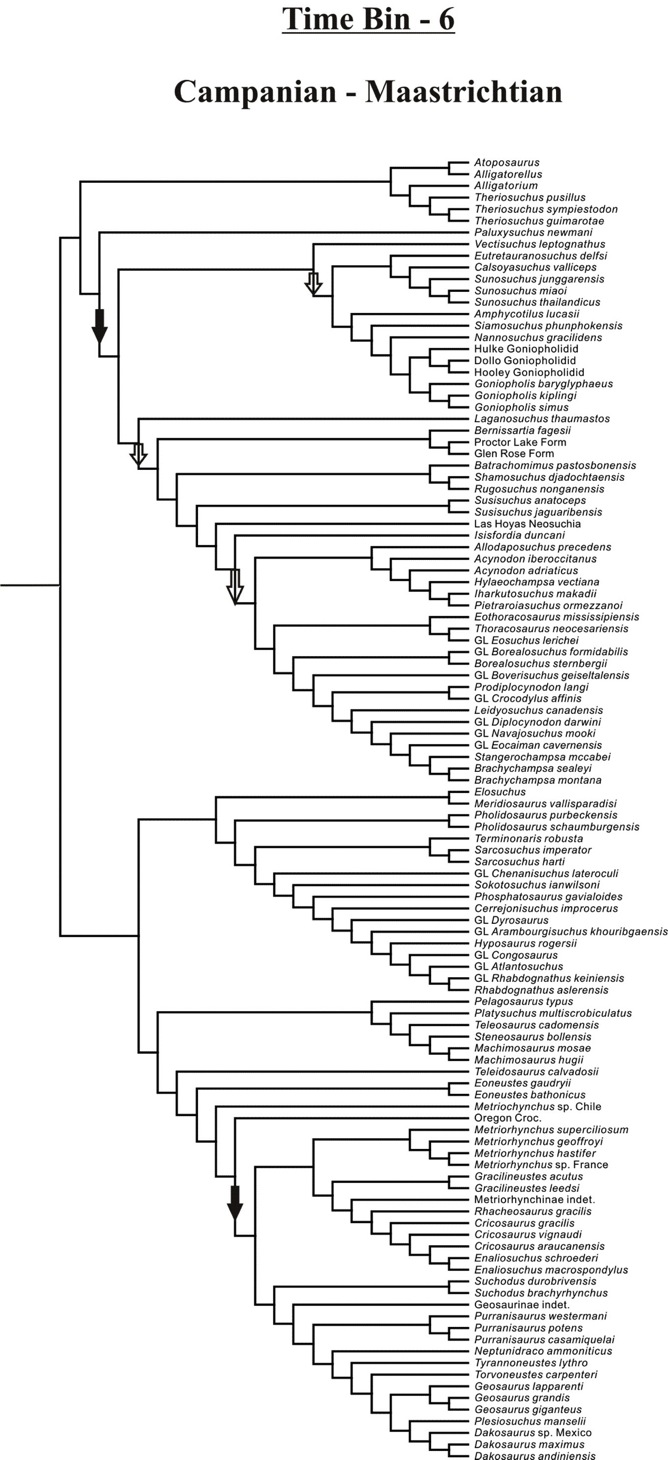

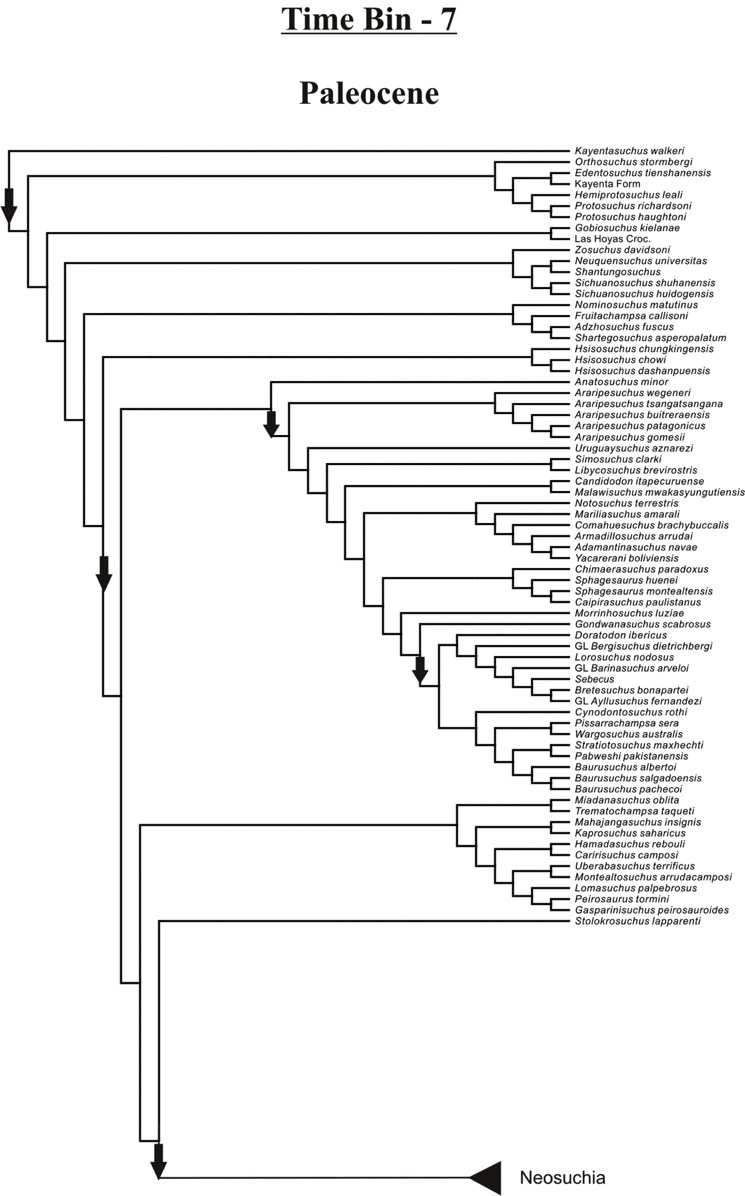

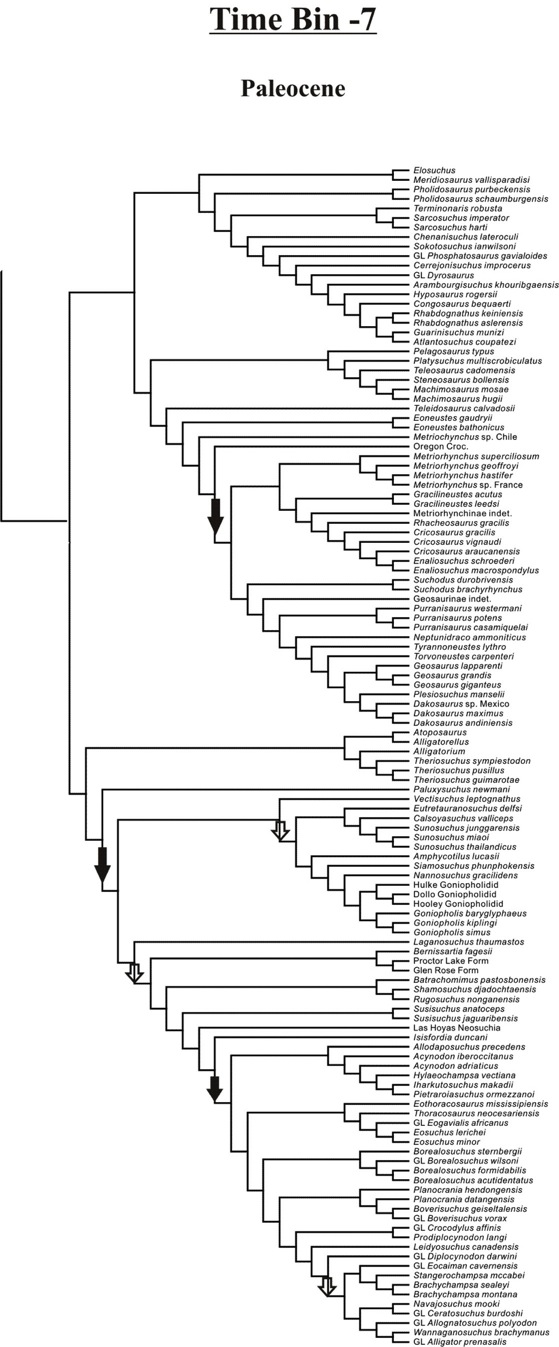

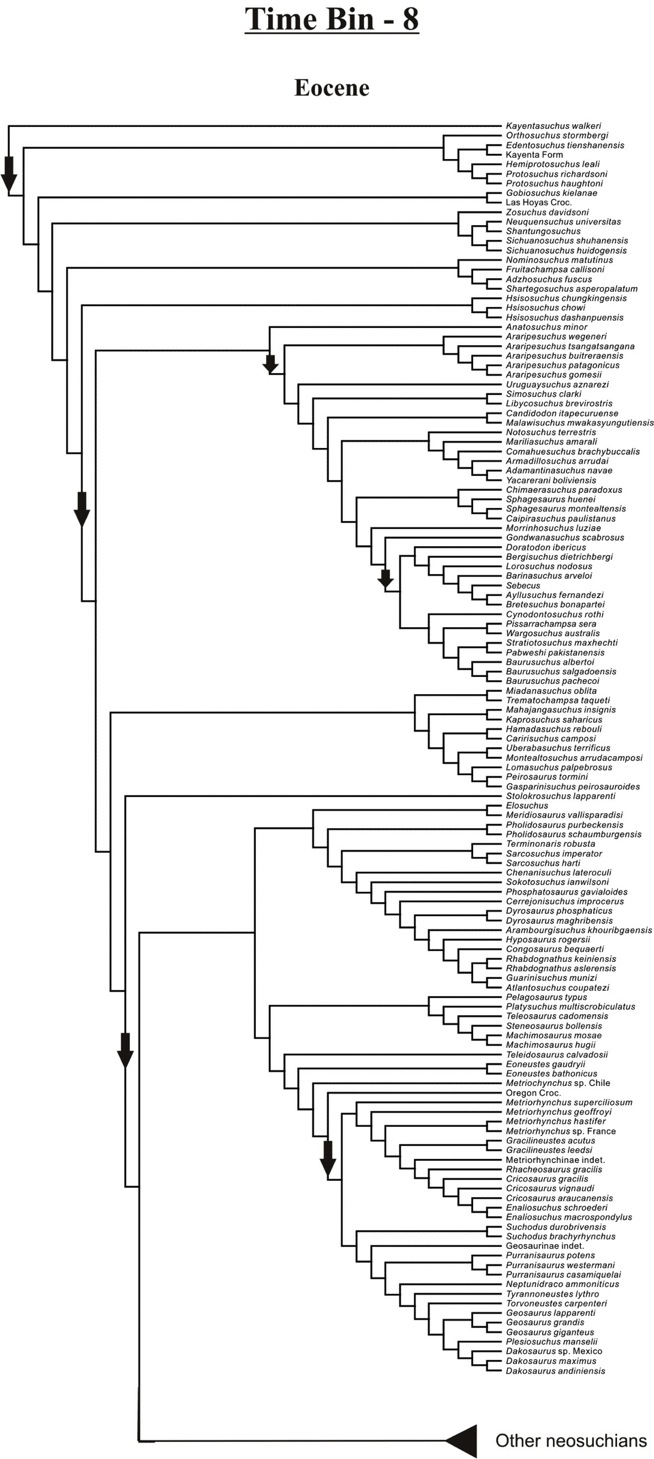

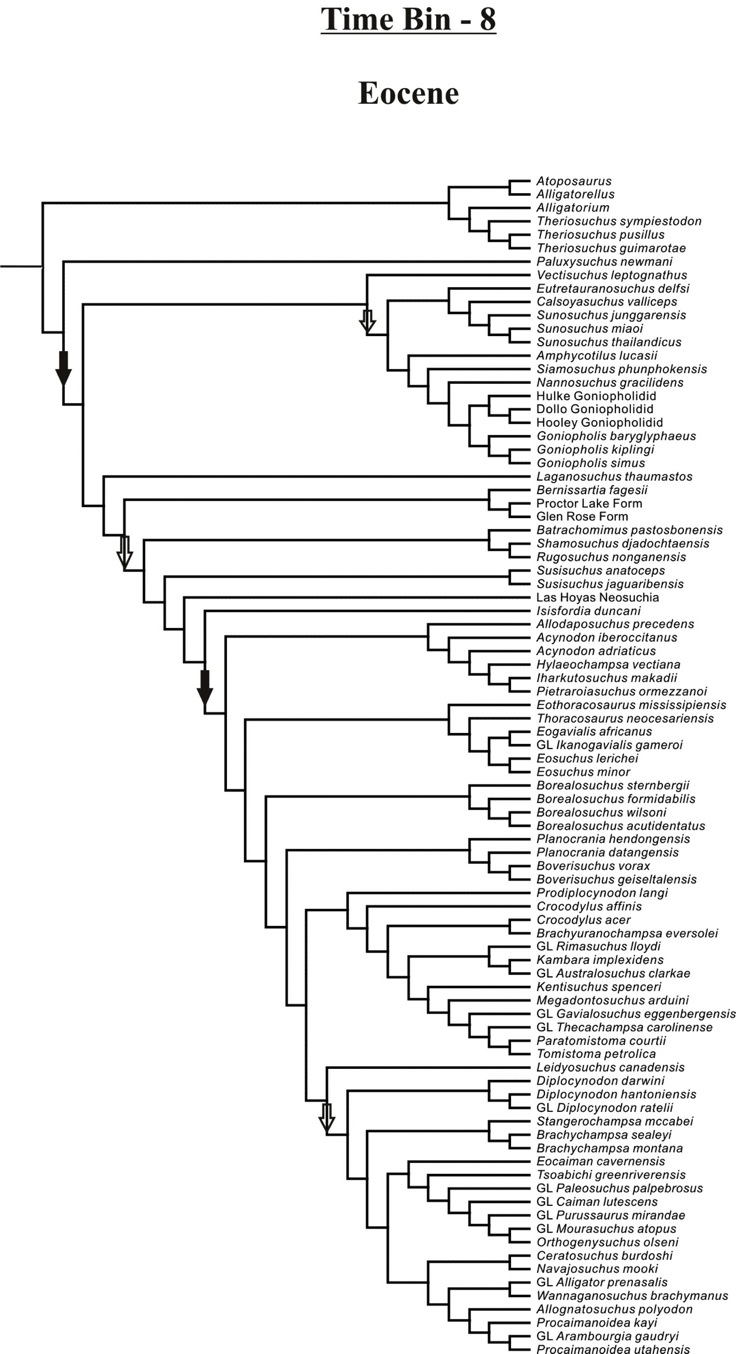

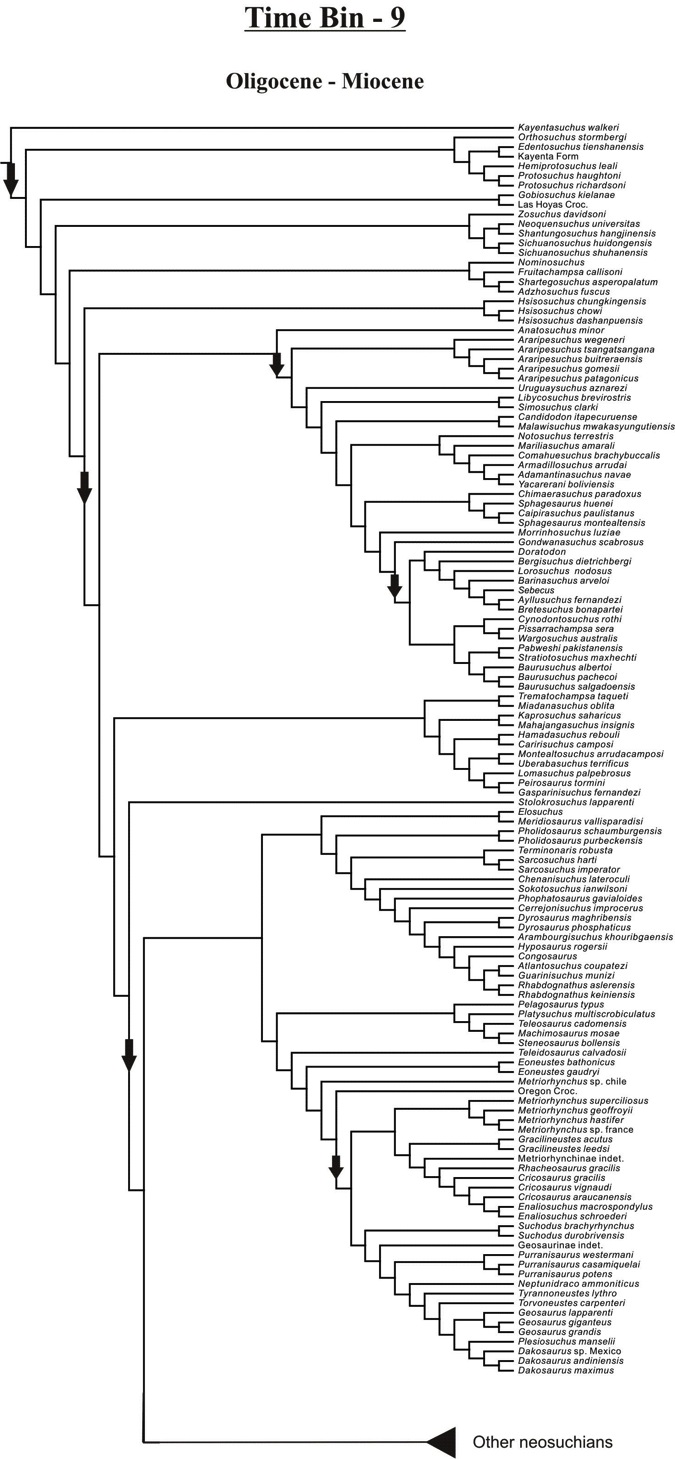

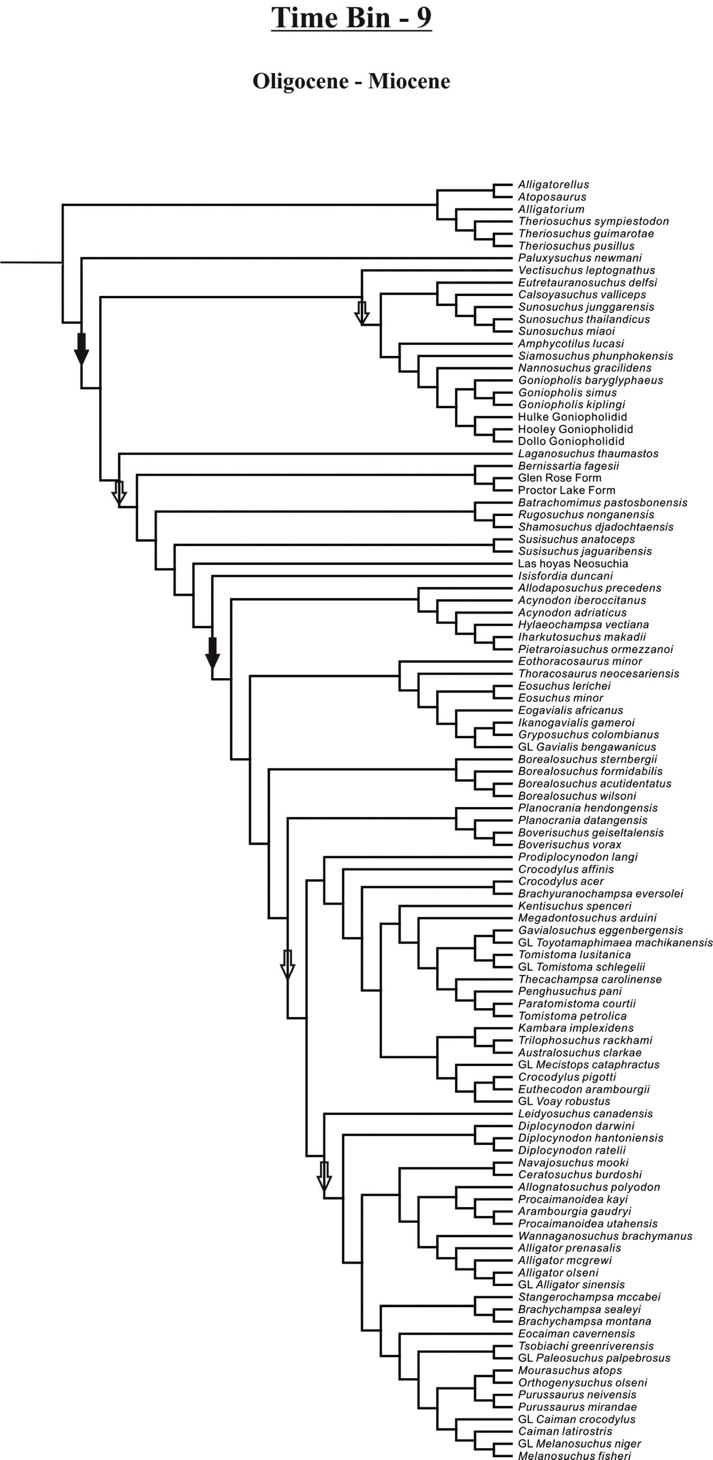

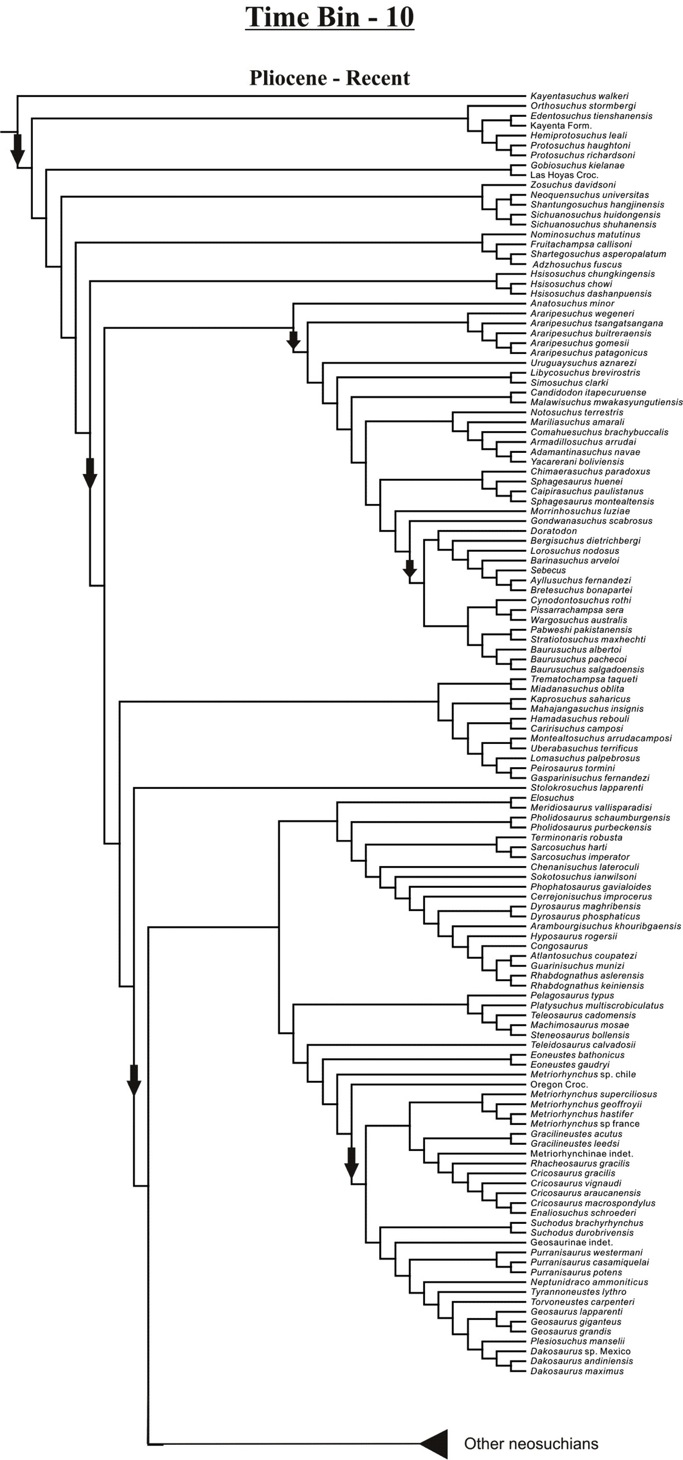

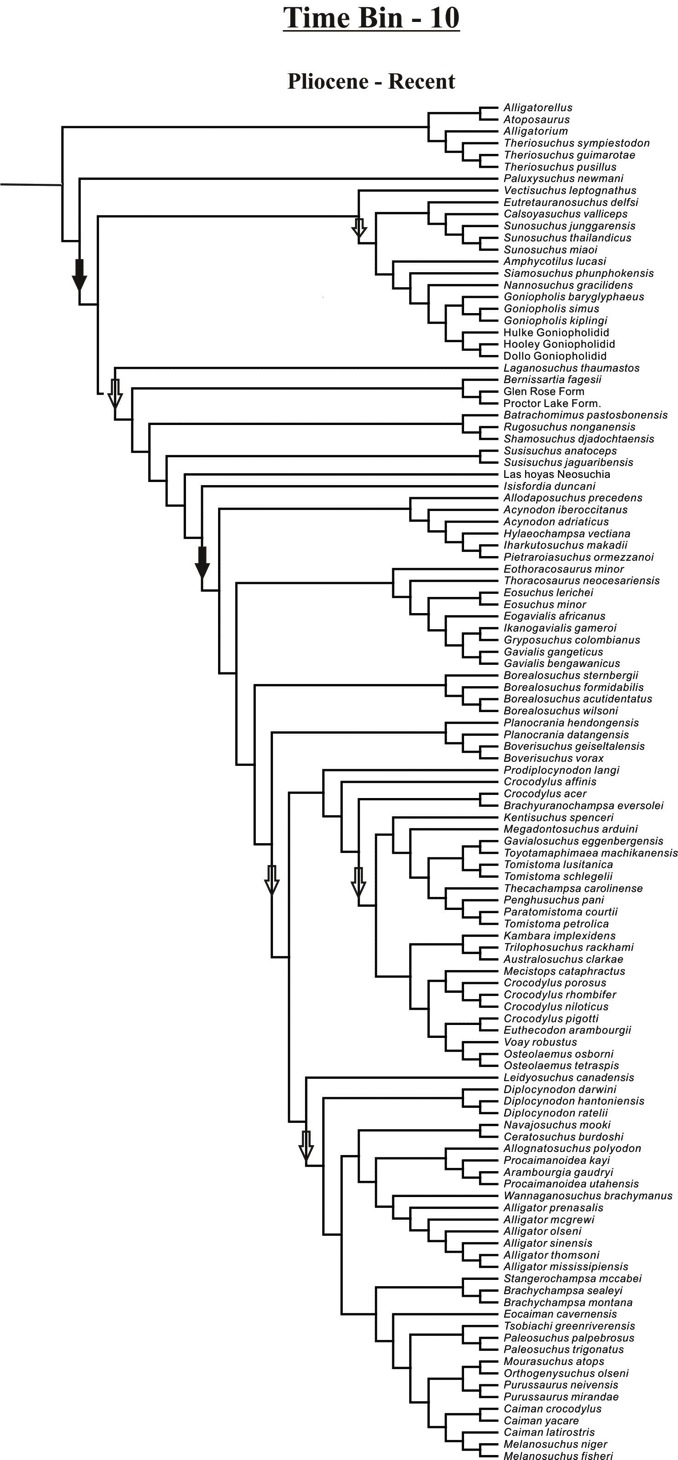


**Additional References**

Andrade, M. B., Edmonds, R., Benton, M. J.; Schouten, R. 2011. A new Berriasian species of *Goniopholis* (Mesoeucrocodylia, Neosuchia) from England, and a review of the genus. *Zoological Journal of the Linnean Society*, 163:S 66–S108

Brochu CA. 2012 Phylogenetic relationships of Paleogene ziphodont eusuchians and the status of *Pristichampsus* Gervais, 1853. *Earth Environ. Sci. Trans. R. Soc. Edinb*. **103(3-4)**: 521-550. (doi: 10.1017/S1755691013000200)

Bronzati, M., Montefeltro, F. C. & Langer, M. C. 2012. A species-level supertree of Crocodyliformes. *Historical* *Biology*, Published online

Goloboff, P. A.; Farris, J. S.; Nixon, K. C. 2008. TNT, a free program for phylogenetic analysis. Cladistics 24: 774-786.

Montefeltro, F. C.; Larsson, H. C. E.; Franca, M. A. G.; Langer, M. C. 2013. A new Neosuchian with Asian affinities from the Jurassic of northeastern Brazil. *Naturwissenschaften*. 100(9) 835:841.

Pol, D. & Escapa, I. H. 2009. Unstable taxa in cladistic analysis: identification and the assessment of relevant characters. *Cladistics.* 25(5): 515-527.

Tarver, J. E. & Donoghue. 2011. The Trouble with Topology: Phylogenies without Fossils Provide a Revisionist Perspective of Evolutionary History in Topological Analyses of Diversity. *Systematic Biology.* 60(5): 700-712.

Turner, A.H. & Buckley, G.A. 2008. *Mahajangasuchus insignis* (Crocodyliformes: Mesoeucrocodylia) cranial anatomy and new data on the origin of the eusuchian-style palate. *Journal of Vertebrate Paleontology* 28: 382–408.

Turner, A. H. & Sertich, J. W. 2010. Phylogenetic history of *Simosuchus clarki* (Crocodyliformes: Notosuchia) from the Late Cretaceous of Madagascar". *Journal of Vertebrate Paleontology* 30 (6, Supplement): 177–236.
